# Catalytic enantioselective intramolecular Tishchenko reaction of *meso*-dialdehyde: synthesis of (*S*)-cedarmycins†

**DOI:** 10.1039/d1ra00915j

**Published:** 2021-03-22

**Authors:** Ismiyarto, Nobuki Kishi, Yuki Adachi, Rui Jiang, Takahiro Doi, Da-Yang Zhou, Kaori Asano, Yasushi Obora, Takayoshi Suzuki, Hiroaki Sasai, Takeyuki Suzuki

**Affiliations:** The Institute of Scientific and Industrial Research, Osaka University Mihogaoka, Ibaraki Osaka 567-0047 Japan suzuki-t@sanken.osaka-u.ac.jp; Department of Chemistry and Materials Engineering, Faculty of Chemistry, Materials, and Bioengineering, Kansai University Suita Osaka 564-8680 Japan

## Abstract

The first successful example of a catalytic enantioselective intramolecular Tishchenko reaction of a *meso*-dialdehyde in the presence of a chiral iridium complex is described. Chiral lactones were obtained in good yields with up to 91% ee. The obtained enantioenriched lactones were utilized for the first synthesis of (*S*)-cedarmycins A and B.

## Abstract 2

The first successful example of a catalytic enantioselective intramolecular Tishchenko reaction of a *meso*-dialdehyde in the presence of a chiral iridium complex is described.

## Graphical abstract

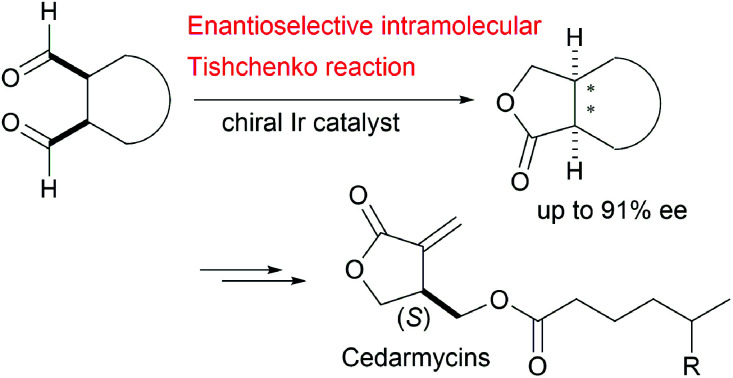

The catalytic dimerization of aldehydes giving the corresponding esters was first discovered by Claisen in 1887,^1^ and is now well known as the “Tishchenko reaction” (Scheme 1).

**Scheme 1:**
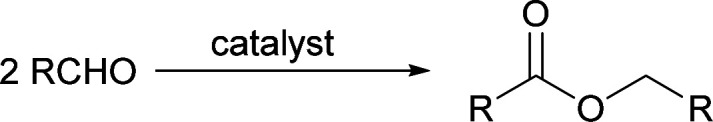
Tishchenko reaction.

Claisen's method utilizing sodium alkoxides, however, could only be applied to nonenolizable aldehydes like benzaldehyde, because enolizable aldehydes undergo aldol reactions when treated with strong bases such as sodium alkoxides.^1^ In 1906, a Russian chemist, Tishchenko, reported that aluminum alkoxides were superior to sodium alkoxides in the reaction, because they were more Lewis acidic and less basic.^2^ The transformation of acetaldehyde into ethyl acetate is the representative application of the Tishchenko reaction in the chemical industry.^3^ So far, a number of homogeneous catalysts exhibiting high catalytic performance for the Tishchenko reaction have been developed to compensate for the drawback of the classical aluminum alkoxide catalysts.^4^ In 2005, we reported a mild Tishchenko dimerization using a Ir catalyst.^5^ The reaction proceeds at room temperature and is effective with a wide range of aldehydes. We also reported the enantioselective oxidative lactonization^6^ of *meso*-diols for the preparation of chiral lactones.^7^ Although asymmetric aldol-Tishchenko reactions^8^ for chiral 1,3-diols are known, there is no report of an asymmetric intramolecular Tishchenko reaction of *meso*-dialdehydes for chiral lactones. We envisaged that the asymmetric borrowing hydrogen methodology^9^ could be applied to achieve an enantioselective intramolecular Tishchenko reaction of *meso*-dialdehydes (Scheme 2).

**Scheme 2:**
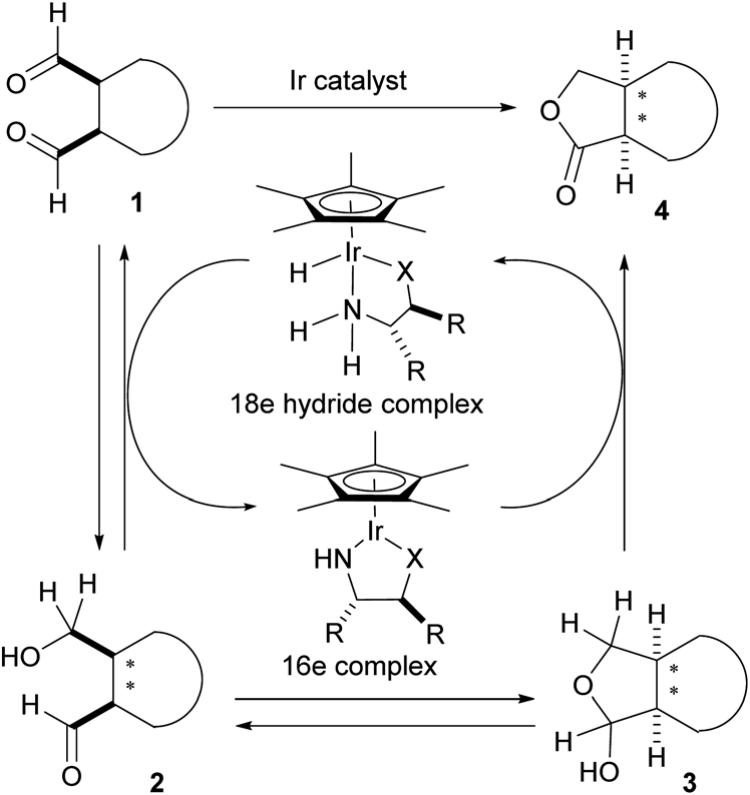
Strategies for enantioselective intramolecular Tishchenko reaction.

The initial enantioselective reduction of *meso*-dialdehyde 1 could occur by a chiral 18 electron Ir hydride complex to afford chiral hydroxy aldehyde 2. The hydroxy aldehyde-lactol equilibrium would generate lactol 3. Finally, irreversible oxidation of 3 would produce the desired lactone 4.

With this hypothesis in mind, we began the investigation of the intramolecular Tishchenko reaction using *o*-phthalaldehyde as a model substrate (Table 1). When a mixture of *o*-phthalaldehyde (6a) in CH_2_Cl_2_ containing the Ir complex 5a,^10^^*i*^PrOH, and K_2_CO_3_ (6a : 5a : ^*i*^PrOH : K_2_CO_3_ = 100 : 1 : 20 : 20 mol ratio) was stirred at 30 °C for 7 h, lactone (7a) was obtained in 97% yield (entry 1). The reaction did not proceed at all in the absence of Ir catalyst (entry 2). Interestingly, the reaction without the additional hydrogen donor, ^*i*^PrOH, also gave the lactone, but the yield was low (entry 3). To enhance the reactivity, the addition of a base was necessary.^5*a*^ Without base or with a lower amount of base, the reaction proceeded in less than 28% yield (entries 4, 5). The reaction of 2,3-naphthalene dicarboxaldehyde 6b also proceeded similarly under optimized conditions (entry 6).

**Table 1:** Intramolecular Tishchenko reaction of aromatic dialdehydes^a^

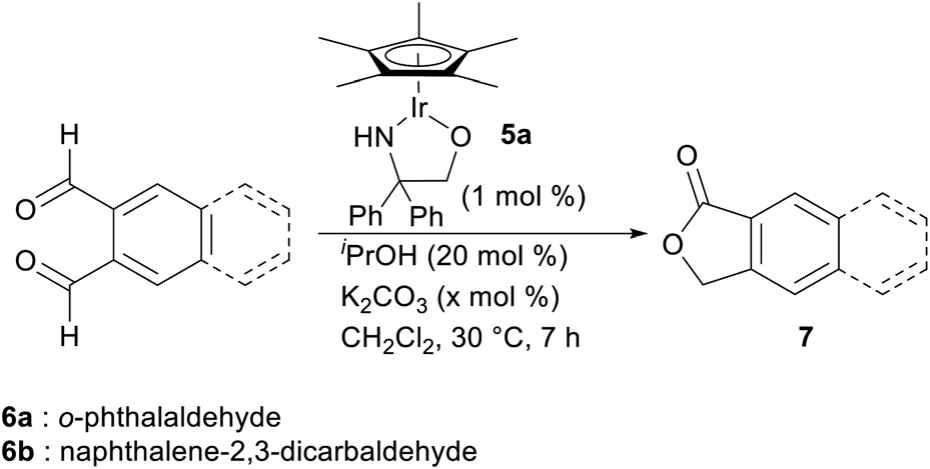					
Entry	Aldehyde	Ir (5a)	^*i*^PrOH	K_2_CO_3_ (*x* mol%)	Yield (%)
1	6a	+	+	20	97
2	6a	—	+	20	0
3	6a	+	—	20	24
4	6a	+	+	—	6
5	6a	+	+	10	28
6	6b	+	+	20	97

a All the reactions were carried out on a 0.15 mmol scale of 6.

Encouraged by these results, we next examined the enantioselective intramolecular Tishchenko reaction of a *meso*-dialdehyde. With the utility of the product in mind, we selected the *meso*-aldehyde 10 as a model substrate (Scheme 3). The requisite aldehyde was prepared in 3 steps from *cis*-3-cyclobutene-1,2-dimethanol 8.^11^ Protection of the diol with diphenyldiazomethane followed by dihydroxylation gave the *anti*-diol 9 in 58% yield as the major isomer. The relative configuration of 9 was unambiguously determined by the crystalline sponge (CS) method.^12^ Oxidative cleavage^13^ of 9 by silica gel-supported NaIO_4_ gave the desired *meso*-dialdehyde 10.

**Scheme 3:**
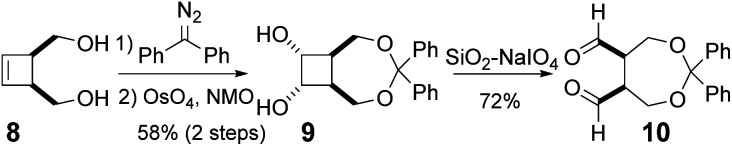
Synthesis of *meso*-dialdehyde 10.

With the *meso*-dialdehyde 10 in hand, we investigated the enantioselective intramolecular Tishchenko reaction using a chiral Ir complex. After screening the catalysts and reaction conditions, we were pleased to find that the enantioselective intramolecular Tishchenko reaction was realized for the first time. Thus, treatment of 10 with Cp*Ir [(*R*,*R*)-Tsdpen] (5b;^14^ TsDPEN = *N*-(*p*-toluenesulfonyl)-1,2-diphenylethylenediamine) (10 mol%) and ^*i*^PrOH (20 mol%) in the presence of K_2_CO_3_ (40 mol%) in CH_2_Cl_2_ at 30 °C for 24 h provided the desired 11 in 83% yield with 78% ee (Table 2, entry 1). The reaction in acetonitrile proceeded quantitatively, but the enantioselectivity was diminished (entry 2). The reaction in the presence of K_3_PO_4_ slightly increased the chemical yield and ee (entry 3). Finally, the addition of a phosphoric acid,^15^ (PhO)_2_PO_2_H, increased the enantioselectivity (91% ee, entry 4). Similar positive effects of the phosphoric acid on the enantioselectivity were also observed with 10 mol% of chiral phosphoric acids such as TRIP (TRIP = 3,3′-bis(2,4,6-triisopropylphenyl)-1,1′-binaphthyl-2,2′-diylhydrogen phosphate). However (*R*)-TRIP and (*S*)-TRIP gave the same enantioselectivity.

**Table 2:** Enantioselective intramolecular Tishchenko reaction of *meso*-dialdehyde 10^a^

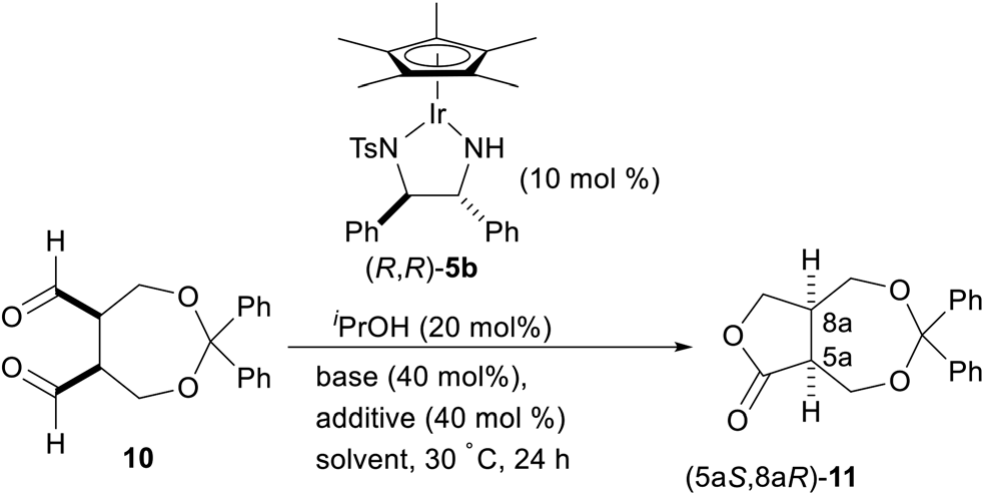					
Entry	Solvent	Base	Additive	Yield^b^ (%)	Ee^c^ (%)
1	CH_2_Cl_2_	K_2_CO_3_	None	83	78
2	CH_3_CN	K_2_CO_3_	None	>99	68
3	CH_2_Cl_2_	K_3_PO_4_	None	87	80
4	CH_2_Cl_2_	K_2_CO_3_	(PhO)_2_PO_2_H	78	91

a All the reactions were carried out on a 0.15 mmol scale of 10.

b Isolated yield.

c Determined by chiral HPLC.

The absolute configuration of 11 was unambiguously determined by single crystal X-ray crystallographic analysis using the optically pure lactone obtained by recrystallization (Fig. 1).

**Fig. 1:**
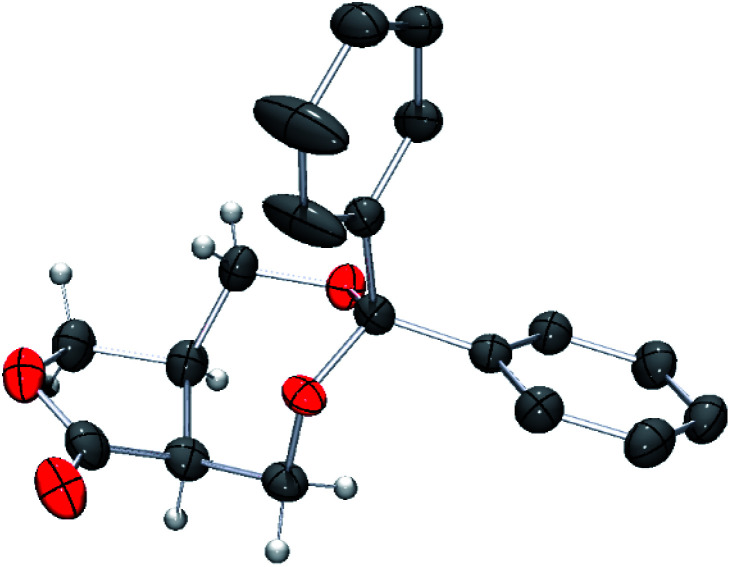
POV-ray depiction of (5a*S*,8a*R*)-11. Thermal ellipsoid is drawn at the 50% probability level. H atoms on benzene rings are omitted for clarify.

To compare the enantioselective intramolecular Tishchenko reaction with the enantioselective oxidative lactonization,^6^ the corresponding diol 12 was treated with the same catalyst in the presence of acetone as an oxidant, to afford the desired lactone 11 in 92% yield and 79% ee with (5a*S*,8a*R*) configuration (Scheme 4). The addition of (PhO)_2_PO_2_H did not improve the enantioselectivity in this reaction system (87%, 78% ee).

**Scheme 4:**
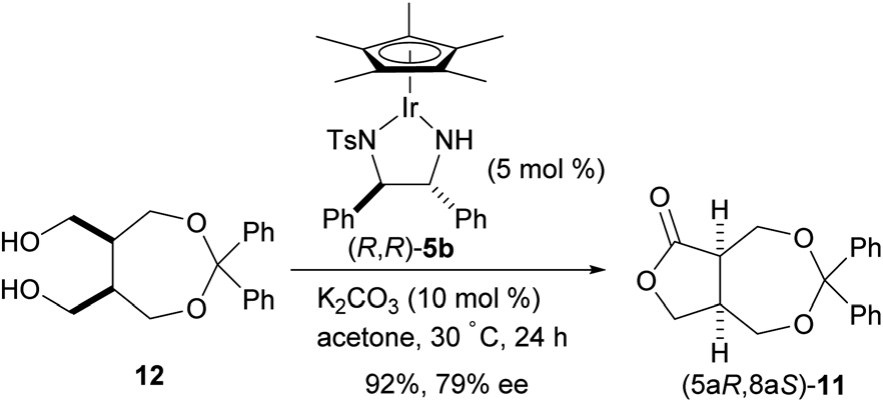
Enantioselective oxidative lactonization of *meso*-diol 12.

These results show that both enantiomers of the lactone can be prepared using the same catalyst by selecting the appropriate reaction system (Scheme 5). The enantioselective intramolecular Tishchenko reaction and the enantioselective oxidative lactonization are complementary, indicating both reactions pass through the same transition state.

**Scheme 5:**
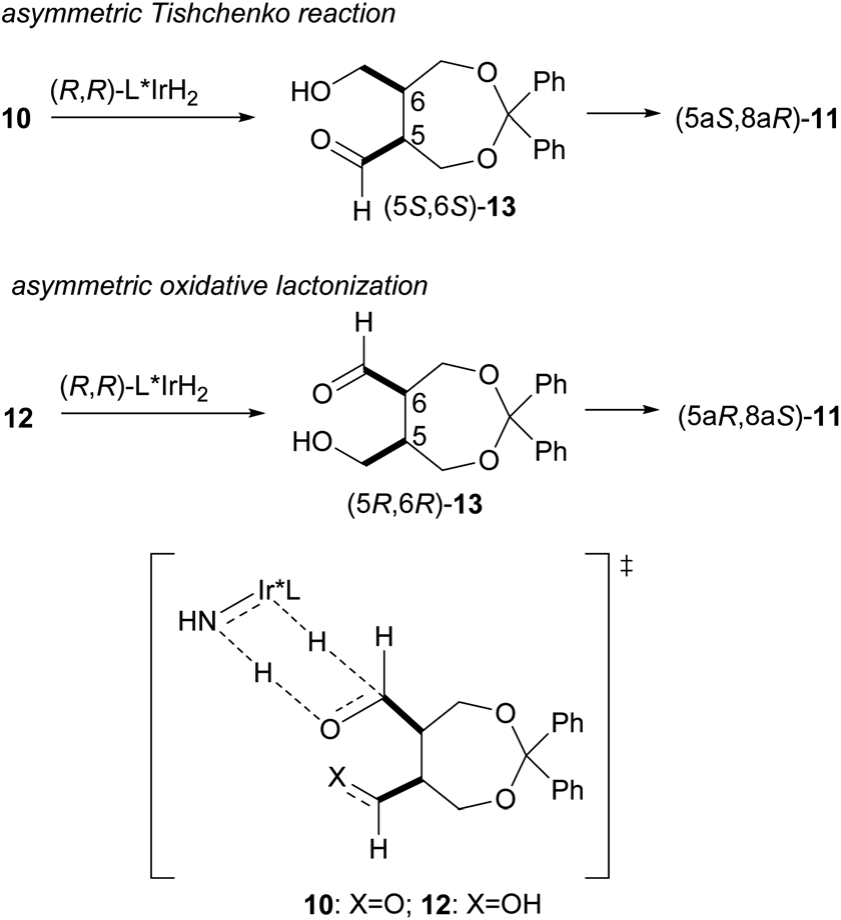
Comparison of enantioselective intramolecular Tishchenko reaction with enantioselective oxidative lactonization.

In this reaction, we used 16 electron Ir complex 5b and ^*i*^PrOH to generate the 18 electron Ir hydride complex. To obtain information on the catalyst, cold spray ionization mass spectrometry (CSI-MS)^16^ was conducted. The sample prepared from 5b and iPrOH gave a prominent peak at *m*/*z* 695 corresponding to the 18 electron Ir hydride complex, Cp*IrH[TsNCHPhCHPhNH_2_] + H^+^. The Ir complex formed from 5b and 1 equiv. of (PhO)_2_PO_2_H in ^*i*^PrOH exhibited a peak at *m*/*z* 965 due to Cp*IrH[TsNCHPhCHPhNH_2_][OPO(OPh)_2_] + Na^+^. These results indicate that Cp*Ir, TsDPEN, and phosphoric acid contribute to the formation of an efficient asymmetric environment.^15^

Having succeeded in the first intramolecular enantioselective Tishchenko reaction of *meso*-dialdehyde, we then applied our method to the synthesis of several natural products. Cedarmycins were isolated in 2001 by the Frumai and Igarashi groups from a plant called *Cryptomeria japonica*.^17^ Cedarmycins exhibit antibiotic activity with cedarmycin A showing potent activity against candida glabrata IFO 0622, comparable to amphotericin B. To date, two groups have reported different methods for the racemic synthesis of the cedarmycins.^18^ However, no reports on the catalytic asymmetric synthesis or the absolute configurations of these compounds have been published. As shown in Scheme 6, the deprotection of 11 under hydrogenolysis conditions afforded the desired *cis*-diol 14. Sequential double acylation/elimination of 14 proceeded in the presence of DBU to give cedarmycins A (15a) and B (15b) in 76% and 85% yields, respectively. By comparison of their optical rotations, the absolute configurations of cedarmycins A and B were determined to be (*S*). It should be noted that cleavage of the acetal moiety of 11 by acid hydrolysis caused partial racemization of 15. The racemization likely occurred by the recyclization of *cis*-14 by the nucleophilic attack of the γ-hydroxy group under acidic conditions.

**Scheme 6:**
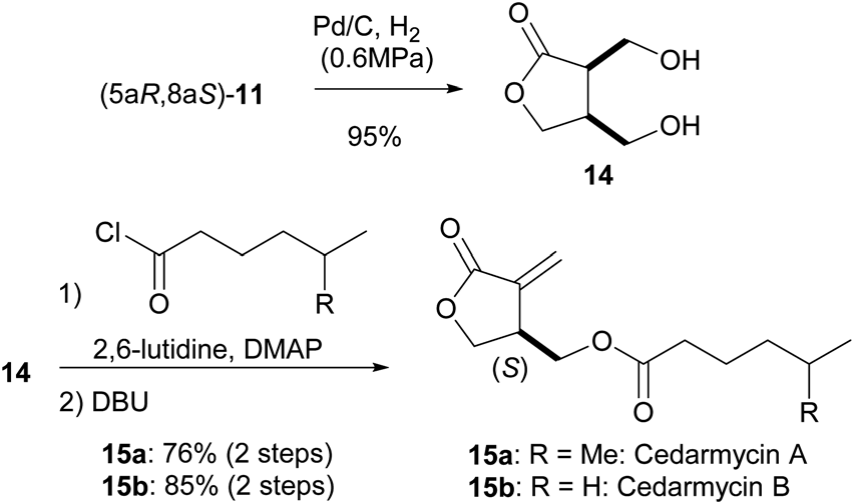
Catalytic enantioselective synthesis of cedarmycins A and B.

In conclusion, we have achieved the first enantioselective intramolecular Tishchenko reaction of *meso*-dialdehydes and applied this methodology to the synthesis of natural products. Chiral lactones are useful chiral building block for the organic synthesis. The direct conversion of 1,4-dialdehydes to γ-lactones is attractive from the view point of environmentally benign redox neutral process.^19^ Additional studies on the substrate scope and reaction mechanism are currently in progress in our laboratory.

## Conflicts of interest

There are no conflicts to declare.

## Supplementary Material

RA-011-D1RA00915J-s001

RA-011-D1RA00915J-s002
