# Supplementary material for: Catalytic enantioselective intramolecular Tishchenko reaction of meso-dialdehyde: synthesis of (S)-cedarmycins
Source: RSC Adv. 2021 Mar 22;11(19):11606–9. doi: 10.1039/d1ra00915j (PMC8695919; doi:10.1039/d1ra00915j)
Supplement: RA-011-D1RA00915J-s001 [file RA-011-D1RA00915J-s001.pdf]

Electronic Supplementary Material (ESI) for RSC Advances.
This journal is © The Royal Society of Chemistry 2021

Electronic Supplementary Information (ESI):

Catalytic Enantioselective Tishchenko Reaction of meso-Dialdehyde: Synthesis of (*S*)-Cedarmycins

Ismiyarto,[a] Nobuki Kishi,[b] Yuki Adachi,[a] Rui Jiang,[a] Takahiro Doi,[a] Da-Yang Zhou,[a] Kaori Asano,[a] Yasushi Obora,[b] Takayoshi Suzuki,[a] Hiroaki Sasai,[a] and Takeyuki Suzuki*[a]

[a]*The Institute of Scientific and Industrial Research, Osaka University, Mihogaoka, Ibaraki, Osaka 567-0047, Japan.*

[b]*Department of Chemistry and Materials Engineering, Faculty of Chemistry, Materials, and Bioengineering, Kansai University, Suita, Osaka 564-8680, Japan*

E-mail: suzuki-t@sanken.osaka-u.ac.jp

Table of Contents

1. General……S2
2. Experimental Section……S2
3. CSI-MS of the Ir complex……S5
4. Determination of the structure by X-ray Crystallography……S8
5. HPLC Chart……S11
6. NMR Spectra……S14

Experimental Procedures

1.General

Melting points were obtained with a Yanagimoto Micro Melting Point Apparatus and are uncorrected. Infrared (IR) spectra were recorded on a JASCO FT/IR 4100 spectrometer. ^{1}H NMR spectra were recorded on JEOL JNM-ECS400 NMR or JEOL JNM-ECA600 NMR or Bruker Avance III 700 NMR spectrometer. The chemical shifts are reported in ppm on the ***δ*** scale downfield from tetramethylsilane or relative to the residual sovent signals ($CDCl_3$: 7.26 ppm for ^{1}H NMR and 77.16 for ^{13}C NMR, CD_2Cl_2: 5.32 ppm for ^{1}H NMR and 53.84 for ^{13}C NMR, CD_3OD: 3.31 ppm for ^{1}H NMR and 49.00 for ^{13}C NMR), and signal patterns are indicated as follows: s, singlet; d, doublet; t, triplet; m, multiplet; br, broad peak. ^{13}C NMR spectra were measured on a JEOL JNM-ECA600 NMR spectrometer at 151 MHz or Bruker Avance III 176 NMR spectrometer at 176 MHz. APCI or ESI mass spectra were recorded on a THERMO LTQ Orbitrap XL spectrometer. CSI mass spectra were recorded on a Bruker micrOTOF II spectrometer. X-ray crystallographic analyses were conducted on a Rigaku E-AXIS RAPID 191R diffractometer system equipped with a Rigaku FR-E^{++} SuperBright (Cu) X-ray generator or a Rigaku XtaLAB PRO MM007 DW diffractometer system equipped with a MicroMax007HFM-DW(Cu/Mo) X-ray generator and a HyPix-6000HE detector. Optical rotations were measured with JASCO P-2300 polarimeter. HPLC analyses were performed on SHIMADZU HPLC system (SHIMADZU LC20 AD pump and SPD-M20A PDA detector). Anhydrous THF and methanol were purchased from Kanto Chemicals and used without any purification. Other solvents were purified prior to use by standard techniques. 5% Pd/C (N.E.Chemcat NX type) was purchased and used without any purification.

2. Experimental section

Intramolecular Tishchenko Reaction of Aromatic Dialdehyde （Table 1）

The mixture of **6** (0.15 mmol), K_2CO_3 (4.1 mg, 0.03 mmol, 20 mol %), 0.6 M *i*-PrOH in CH_2Cl_2 solution (0.05 mL, 0.03 mmol, 20 mol %), and **5a** (0.81 mg, 0.015 mmol, 1 mol %) in CH_2Cl_2 (1 mL) was stirred at 30 °C for 7 h under Ar atmosphere. The mixture was passed through a short silica gel column (ethyl acetate) to remove the catalyst and concentrated under reduced pressure. Chemical yield was determined using 1,1,2,2-tetrachloroethane as internal standard. The crude mixture was purified by silica gel column chromatography (hexane/ ethyl acetate = 1/1) to give the desired product.

phthalide (7a)[1]

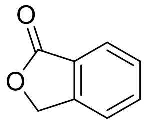

7a

white solid 19.5 mg, 97%.
^{1}H-NMR (700MHz, $CDCl_3$) δ: 7.93 (d, *J* = 7.7 Hz, 1H), 7.70 (td, *J* = 7.4, 1.0 Hz, 1H), 7.55 (td, *J* = 7.5, 0.9 Hz, 1H), 7.52 (dt, *J* = 7.7, 0.9 Hz, 1H), 5.34 (s, 2H).
^{13}C-NMR (176MHz, $CDCl_3$) δ: 171.3, 146.6, 134.1, 129.1, 125.81, 125.78, 122.2, 69.8.

naphtho[2,3-c]furan-1(3H)-one (7b)[2]

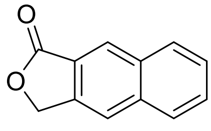

7b

white solid 26.8 mg, 97%.
^{1}H-NMR (700MHz, $CDCl_3$) δ: 8.52 (s, 1H), 8.06 (d, *J* = 8.6 Hz, 1H), 7.95 (d, *J* = 8.2 Hz, 1H), 7.92 (s, 1H), 7.68-7.66 (m, 1H), 7.62-7.60 (m, 1H), 5.50 (s, 2H).
^{13}C-NMR (176MHz, $CDCl_3$) δ: 171.2, 140.1, 136.4, 133.2, 130.1, 129.2, 128.3, 127.2, 127.1, 123.5, 121.0, 69.8.

(1*R*,7*S*,8*S*,9*R*)-4,4-diphenyl-3,5-dioxabicyclo[5.2.0]nonane-8,9-diol (9)

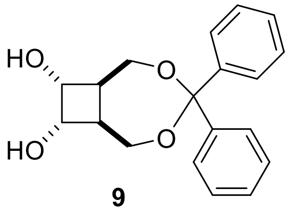

9

cis-3-Cyclobutene-l,2-dimethanol **8**[3] (1.385 g, 12.1 mmol) and diphenyl diazomethane (2.361 g, 12.1 mmol) in 96 mL of 1,2-dichloroethane were slowly added to a solution of 2,3-dichloro-5,6-dicyano-1,4-benzoquinone (DDQ) (2.758 g, 12.1 mmol) in 55 mL of 1,2-dichloroethane at room temperature. The mixture was stirred for 1 h and the reaction mixture was concentrated in vacuo. The concentrate was dissolved in toluene and the solution was washed with saturated $NaHCO_3$. The organic layer was passed through on short column silica gel and concentrated in vacuo in order to give crude acetal product as a yellow oil (3.378 g crude product). To a cooled (0 °C) solution of crude acetal (12.1 mmol) in acetone–H_2O (10:1, 125 mL) was added *N*-methylmorpholine-*N*-oxide (4.27 g, 36.5 mmol) and a solution of osmium tetroxide in ^{t}BuOH (0.04 M, 15,2 mL, 0.607 mmol). The reaction mixture was allowed to warm to ambient temperature over 10 min and attired at 30 °C for 30 min and the reaction was quenched by the addition of saturated aquous $Na_2S_2O_3$ 7.18 g (36.5 mmol). After stirring for 20 min, the layers were separated and the aqueous layer was extracted with AcOEt. The combined organic layers were washed with brine, dried over Na_2SO_4, and concentrated in vacuo. The resulting oil was purified by flash chromatography (30:70 EtOAc–hexanes) to afford **9** as a white solid (2.21 g, 58% in two steps).

MP 178°C
^{1}H-NMR (700MHz, $CDCl_3$) δ: 7.59-7.55 (m, 4H), 7.28 (t, *J* = 7.7 Hz, 4H), 7.21-7.20 (m, 2H), 4.17 (s, 2H), 3.82 (dd, *J* = 12.9, 6.5 Hz, 2H), 3.65-3.63 (br m, 2H), 3.11 (d, *J* = 3.4 Hz, 2H), 2.48-2.48 (m, 2H).

^{13}C-NMR (176MHz, $CDCl_3$) δ: 144.1, 143.2, 128.3(2C), 127.7(2C), 126.4(4C), 126.3(4C), 105.0(2C), 68.5(2C), 62.1 (2C).
IR(KBr): 3277, 3023,2939,1054,1031,1012cm^{-1}
APCI-HRMS. Calcd for $C_{19}H_{20}O_4$ $[M+Na]^+$: 335.1259. Found: 335.1254.

Synthesis of *meso*-Dialdehyde 10

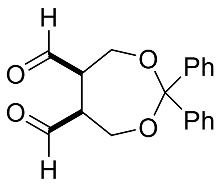

10

To a vigorously stirred suspension of silica gel-supported $NaIO_4$[4] (666 mg, 0.448mmol, 2 equiv) was added a solution of the diol **9** (70 mg, 0.224 mmol) in CH_2Cl_2 (2.24 mL, 0.1 M). The reaction was monitored by TLC until disappearance of the starting material (generally 10-30 min). The mixture was filtered through a sintered glass funnel, and the silica gel was thoroughly washed with CH_2Cl_2 to total volume 10 mL. The chemical yield (72%) was determined by ^{1}H-NMR using tetrachloroethane as an internal standard. For Tishchenko reaction in the table 2, the residue obtained by evaporation of the CH_2Cl_2 solution was used quickly by addition of CH_2Cl_2 or CH_3CN. The sample for the NMR measurement was prepared in the same manner except for using CD_2Cl_2 instead of CH_2Cl_2.

^{1}H-NMR (700MHz, CD_2Cl_2) δ: 9.75 (s, 2H), 7.56 (d, *J* = 7.3 Hz, 2H), 7.47 (d, *J* = 7.3 Hz, 2H), 7.31 (t, *J* = 7.7 Hz, 2H), 7.27 (t, *J* = 7.5 Hz, 2H), 7.25 (t, *J* = 7.3 Hz, 1H), 7.22 (t, *J* = 7.3 Hz, 1H), 4.12 (s, 4H), 3.01 (s, 2H).
^{13}C-NMR (176MHz, CD_2Cl_2) δ: 200.3 (2C), 143.3, 143.1, 128.57 (2C), 128.55 (2C), 128.20, 128.16, 126.5 (2C), 126.3 (2C), 104.7, 61.0 (2C), 52.8 (2C).
IR(KBr): 1730 cm^{-1}
ESI-HRMS. Calcd for $C_{19}H_{18}O_4$ $[M+Na]^+$: 333.1103. Found: 333.1097.

Enantioselective Tishchenko reaction of 10 (Table 2, entry 4)
To a 0.14 M solution of **10** in CH_2Cl_2 (0.46 mL, 0.0644 mmol) was added **5b** (4.5mg, 0.0065mmol, 10 mol %), K_2CO_3 (3.6 mg, 0.0261 mmol, 40 mol %), $(PhO)_2PO_2H$ (6.4 mg, 0.0256 mmol, 40 mol %) and 0.6 M *i*-PrOH in CH_2Cl_2 solution (0.0214 mL, 0.0128 mmol, 20 mol %) and then the mixture was stirred at 30 °C for 24 h under Ar atmosphere. The mixture was passed through a short silica gel column (ethyl acetate) to remove the catalyst and concentrated under reduced pressure. Then crude mixture was purified by silica gel column chromatography (hexane/ ethyl acetate = 85/15) to give the lactone **11** as a white solid (15.6 mg, 78% and 91% ee). The optically pure lactone was prepared from the recrystallization from hexane and ethyl acetate.

(*5aS,8aR*)-3,3-diphenyltetrahydro-1H,6H-furo[3,4-e][1,3]dioxepin-6-one (11)

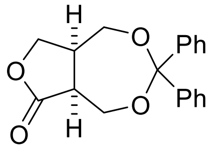

(5a*S*,8a*R*)-**11**

MP 212°C
^{1}H-NMR (700MHz, $CDCl_3$) δ: 7.58-7.53 (m, 4H), 7.32-7.26 (m, 4H), 7.25-7.20 (m, 2H), 4.28 (dd, *J* = 9.5, 6.5 Hz, 1H), 4.23 (d, *J* = 12.7 Hz, 1H), 3.99 (m, 1H), 3.93 (dd, *J* = 12.7, 3.2 Hz, 1H), 3.79 (dd, *J* = 12.5, 4.7 Hz, 1H), 3.68 (dd, *J* = 12.5, 10.8 Hz, 1H), 2.95-2.93 (m, 1H), 2.80-2.80 (m, 1H).
^{13}C-NMR (176MHz, $CDCl_3$)δ: 176.5, 142.9, 142.7, 128.38 (2C), 128.32 (2C), 128.0, 127.9, 126.37 (2C), 126.35 (2C), 104.6, 67.5, 61.9, 59.6, 44.5, 39.1.
IR(KBr): 1777cm^{-1}
APCI-HRMS. Calcd for $C_{19}H_{18}O_4$ $[M+Na]^+$: 333.1103. Found: 333.1097.
$[\alpha]_D^{28}$ –165.1 ° (*c* 1.0, $CHCl_3$, >99% ee).

Preparation of *meso*-Diol 12

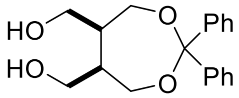

12

To a CH_2Cl_2 solution of *meso*-dialdehyde **10** (99.3 mg, 0.320 mmol) was added MeOH (1.4 mL), then $NaBH_4$ (16mg, 0.422 mmol) was added and stirred at the 30 °C for 24 h. The mixture was quenched by sat. NH_4Cl and extracted with AcOEt, then the organic layer was washed with sat. $NaHCO_3$ and brine, dried over Na_2SO_4. The organic layer was evaporated under reduced pressure to give the desired product **12** as a colorless liquid (88.6 mg, 88% yield).

^{1}H-NMR (600MHz, $CDCl_3$) ^{1}H-NMR ($CDCl_3$) δ: 7.56-7.54 (m, 4H), 7.30-7.26 (m, 4H), 7.21 (t, *J* = 6.9 Hz, 2H), 3.80 (m, 8H), 2.66 (s, 2H), 2.14 (m, 2H).
^{13}C-NMR (150MHz, $CDCl_3$)δ: 143.66, 143.60, 128.20 (2C), 128.1 (2C), 127.58, 127.54, 126.16 (2C), 126.01 (2C), 103.9, 64.5 (2C), 62.6 (2C), 43.24 (2C).
IR(KBr): cm^{-1} 3273 cm^{-1}
APCI-HRMS. Calcd for $C_{17}H_{15}O_2$ $[M+Na]^+$: 337.1416. Found: 337.1411.

Enantioselective Oxidative Lactonization of *meso*-Diol 12
A mixture of Ir complex **5b** (36.3 mg, 0.0525 mmol, 5 mol %), K_2CO_3 (14.5 mg, 0.105 mmol) and diol **12** (329.7 mg, 1.05 mmol) in acetone (5.3 mL) was stirred at 30 °C. After 86 h the resulting solution was passed through a short column chromatography (SiO_2, AcOEt) and evaporated, and the residue was purified by column chromatography (SiO_2, hexane/AcOEt, 1:1) to give **11** (300 mg, 92% as a white solid).
(3*R*,4*S*)-3,4-bis(hydroxymethyl)dihydrofuran-2(3H)-one(*cis*-14)

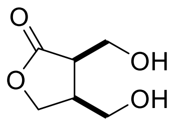

cis-**14**

To a solution of (5a*R*, 8a*S*)-lactone **11** (35 mg, 0.11 mmol) in $CHCl_3$ (0.7 mL) and CF_3CH_2OH (0.7mL) was added 5% Pd/C (24 mg, 0.011 mmol) and stirred at 15 °C for 6 h under H_2 (0.6 MPa). The mixture was filtered by membrane and concentrated under reduced pressure at less than 30 °C. The crude product was purified by silica gel column chromatography (hexane/ ethyl acetate = 1/2, then 0/1) to give the desired product **14** (15.7 mg, 95%, as a colorless oil).

^{1}H-NMR (700MHz, CD_3OD) δ: 4.36 (dd, *J* = 8.7, 7.2 Hz, 1H), 4.26 (dd, *J* = 9.0, 3.8 Hz, 1H), 3.92 (dd, *J* = 11.3, 4.0 Hz, 1H), 3.85 (dd, *J* = 11.3, 7.1 Hz, 1H), 3.80 (dd, *J* = 11.1, 5.0 Hz, 1H), 3.71 (dd, *J* = 11.1, 6.3 Hz, 1H), 2.92-2.91 (m, 1H), 2.81-2.77 (m, 1H).
^{13}C-NMR (175MHz, CD_3OD) δ: 180.0, 71.6, 61.0, 59.2, 45.3, 40.8.
IR(KBr): 3377, 1759cm-1
APCI-HRMS. Calcd for $C_6H_{10}O_4$ $[M+H]^+$: 147.0657. Found: 147.0649.
$[\alpha]_D^{25}$ +34.2 ° (*c* 0.63, AcOEt).

Synthesis of cedarmicine A (15a)

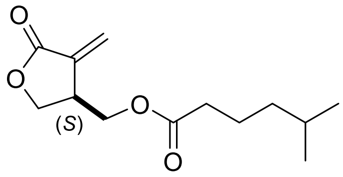

15a:Cedarmycin A

To a solution of **14** (8.4 mg, 0.0575 mmol) in 0.15 mL DCM was added 2,6-lutidine (26.6 µL, 0.23 mmol), DMAP (4.21 mg, 0.0345 mmol) and 5-methylhexanoilchloride[5] (35.5 µL, 0.23 mmol) and stirred at 30 °C for 24 h, then DBU (43 µL, 0.288mmol) was added and stirred for 20 h. The mixture was quenched by NH_4Cl, then extracted with EtOAc. After dried with Na_2SO_4 and evaporated, the crude product was purified by preparative thin layer column chromatography (hexane/ ethyl acetate) to give Cedarmicin A **(15a)** 10.5 mg (76% yield)**.**

^{1}H-NMR (600MHz, $CDCl_3$) δ: 6.39 (d, *J* = 2.7 Hz, 1H), 5.77 (d, *J* = 2.1 Hz, 1H), 4.48 (t, *J* = 8.9 Hz, 1H), 4.25 (dd, *J* = 11.0, 5.5 Hz, 1H), 4.19-4.16 (m, 2H), 3.44-3.44 (m, 1H), 2.31 (t, *J* = 7.6 Hz, 2H), 1.64-1.54 (m, 3H), 1.20-1.17 (m, 2H), 0.88 (d, *J* = 6.2 Hz, 6H).
^{13}C-NMR (150MHz, $CDCl_3$) δ: 173.7, 170.1, 134.7, 124.4, 68.3, 64.9, 38.5, 38.2, 34.5, 27.9, 22.9, 22.6 (2C).
$[\alpha]^{28}_D$ +42.0 (c 0.35, $CHCl_3$, 94% ee), lit. $[\alpha]^{28}_D$ +29.2 (c 1.00, $CHCl_3$) [6].

Cedarmicine B (15b)

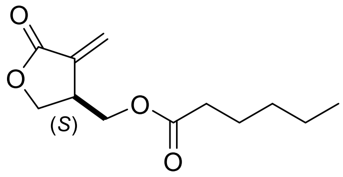

15b: Cedarmycin B

To a solution of **14** (9.5 mg, 0.065 mmol) in 0.15 mL DCM was added 2,6-lutidine (30 µL, 0.26 mmol), DMAP (4.8 mg, 0.039 mmol) and hexanoilchloride (36 µL, 0.26 mmol) and stirred at 30 °C for 20 h, then DBU (49 µL, 0.33 mmol) was added and stirred for 4 h. The mixture was quenched by NH_4Cl, then extracted with EtOAc. After dried with Na_2SO_4 and evaporated, the crude product was purified by preparative thin layer column chromatography (hexane/ ethyl acetate) to give Cedarmicin B **(15b)** 12.5 mg (85% yield)**.**

^{1}H-NMR ($CDCl_3$) δ: 6.38 (1H, d, *J* = 2.7 Hz), 5.76 (1H, d, *J* = 1.4 Hz), 4.48 (1H, t, *J* = 8.9 Hz), 4.25 (1H, dd, *J* = 11.0, 5.5 Hz), 4.19-4.14 (2H, m), 3.46-3.42 (1H, m), 2.32 (2H, t, *J* = 7.6 Hz), 1.64-1.59 (2H, m), 1.36-1.25 (4H, m), 0.90 (3H, t, *J* = 6.5 Hz).
^{1}H-NMR (600MHz, $CDCl_3$) δ: 6.39 (d, *J* = 2.1 Hz, 1H), 5.77 (d, *J* = 2.1 Hz, 1H), 4.48 (t, *J* = 8.9 Hz, 1H), 4.25 (dd, *J* = 11.7, 5.5 Hz, 1H), 4.20-4.15 (m, 2H), 3.45-3.43 (m, 1H), 2.32 (t, *J* = 7.6 Hz, 2H), 1.64-1.60 (m, 2H), 1.32-1.30 (m, 4H), 0.90 (t, *J* = 6.9 Hz, 3H).
^{13}C-NMR (176MHz,$CDCl_3$) δ: 173.7, 170.0, 134.6, 124.4, 68.3, 64.8, 38.2, 34.2, 31.4, 24.7, 22.4, 14.1.
$[\alpha]^{28}_D$ +39.5 (c 0.30, $CHCl_3$, 94% ee), lit $[\alpha]^{28}_D$ +11.7 (c 0.30, $CHCl_3$)[6]

References

1 P.-C. Qian, Y. Liu, R.-J. Song, M. Hu, X.-H. Yang, J.-N. Xiang and J.-H. Li, *Eur. J. Org. Chem.*, 2015, **2015**, 1680-1684.
2 Y. H. Zhang, B. F. Shi and J. Q. Yu, *Angew. Chem. Int. Ed. Engl.*, 2009, **48**, 6097-6100.
3 (a) J. I. Brauman and W. C. Archie, *J. Am. Chem. Soc.*, 1972, **94**, 4262-4265; (b) F. Binns, R. Hayes, S. Ingham, S. T. Saengchantara, R. W. Turner and T. W. Wallace, *Tetrahedron*, 1992, **48**, 515-530; (c) N. Gauvry, C. Comoy, C. Lescop and F. Huet, *Synthesis*, 1999, **1999**, 574-576.
4 Y. L. Zhong and T. K. Shing, *J. Org. Chem.*, 1997, **62**, 2622-2624.
5 R. A. Coats, S. L. Lee, K. A. Davis, K. M. Patel, E. K. Rhoads and M. H. Howard, *J. Org. Chem.*, 2004, **69**, 1734-1737.
6 T. Sasaki, Y. Igarashi, N. Saito and T. Furumai, *J. Antibiot.*, 2001, **54**, 567-572.

3. CSI-MS of the Ir complex.

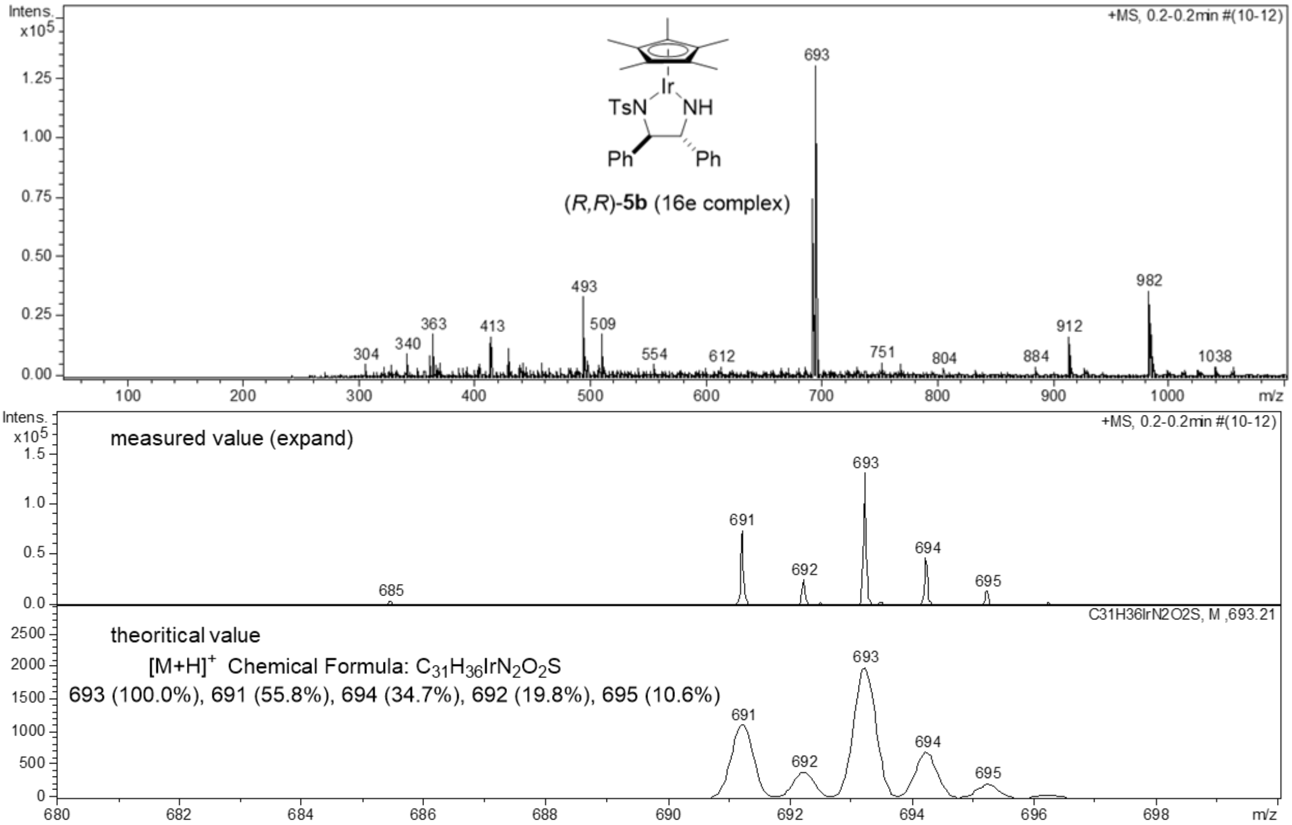

Figure S1. CSI-MS spectrum of Cp*IrTsDPEN in DCM/CH3CN

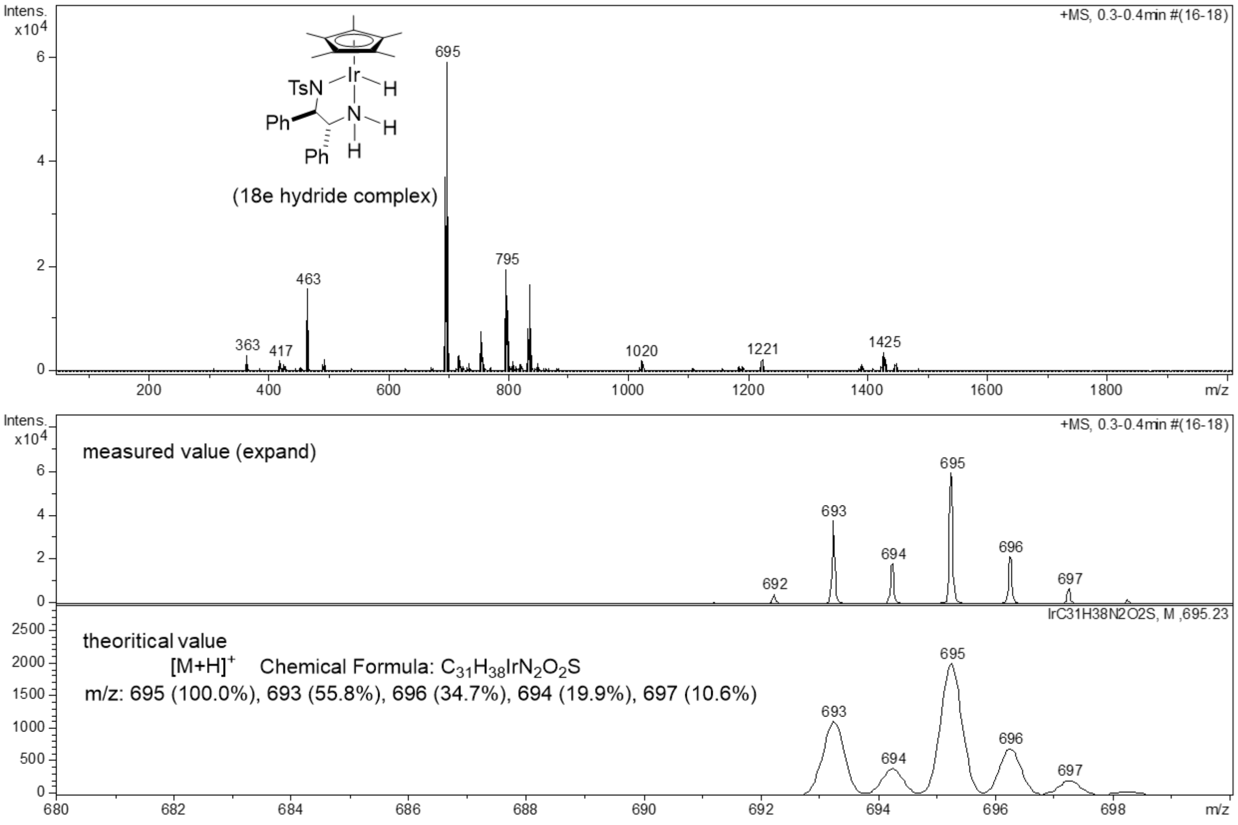

Figure S2. CSI-MS spectrum of Cp*IrTsDPEN in *i*-PrOH.

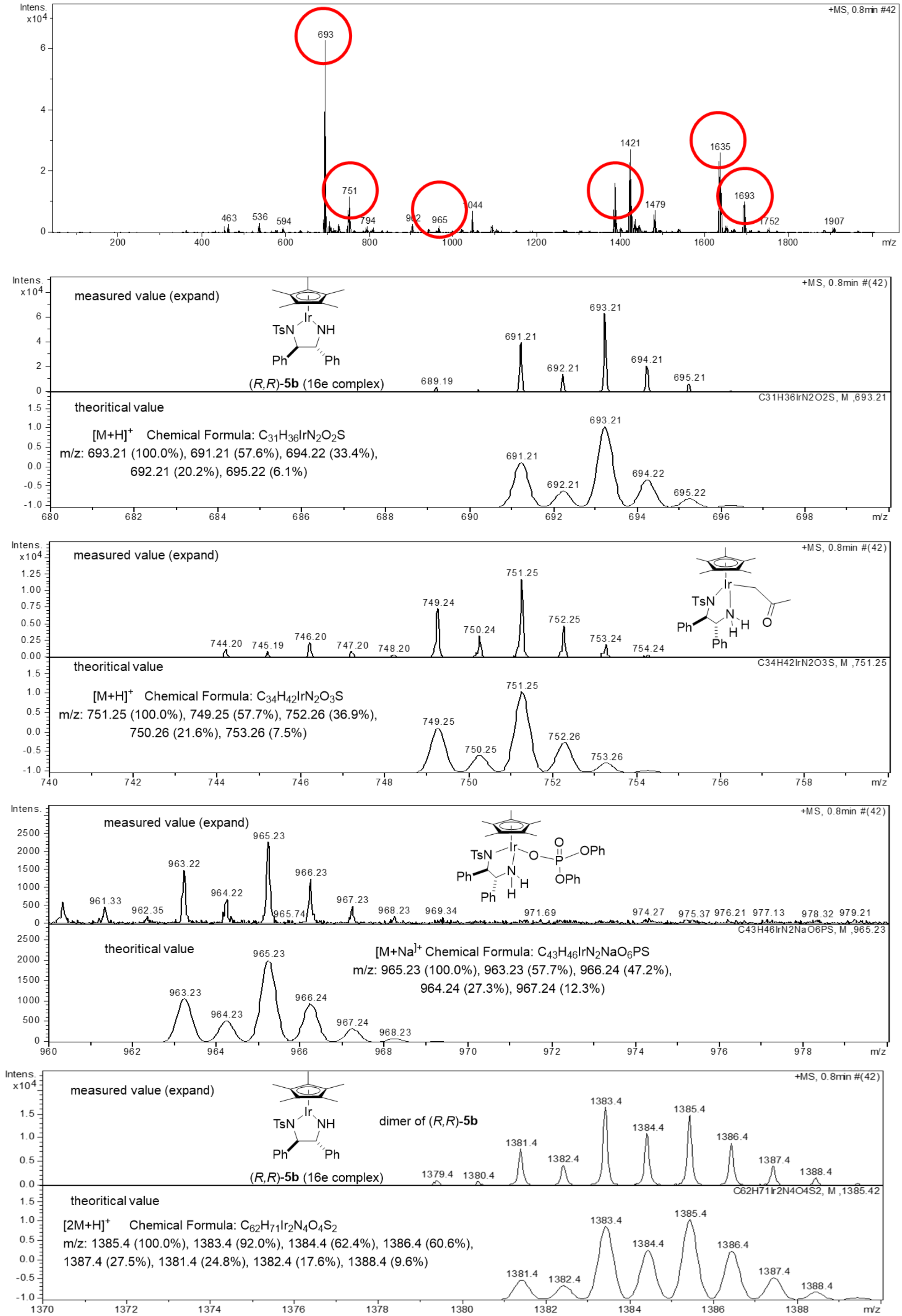

Figure S3. CSI-MS spectrum of a mixture of Cp*IrTsDPEN, $(PhO)_2PO_2H$ (1equiv), K_2CO_3 (1equiv) in DCM/ *i*-PrOH.

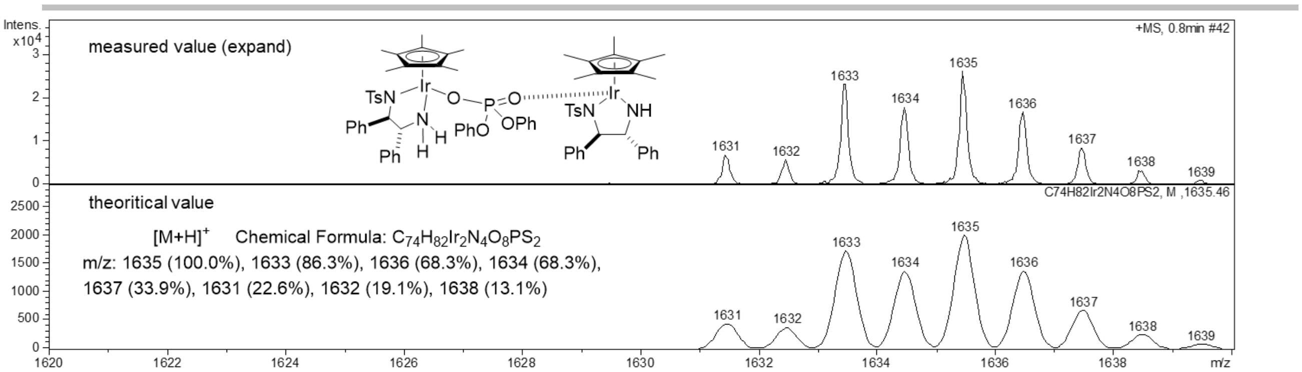
measured value (expand)
+MS, 0.8min #42
TsN
Ir
O
P
O
Ir
Ph
N
H
H
PhO
OPh
TsN
NH
Ph
Ph
Ph
1631
1632
1633
1634
1635
1636
1637
1638
1639
theoritical value
C74H82Ir2N4O8PS2, M ,1635.46
[M+H]+ Chemical Formula: $C_{74}H_{82}Ir_2N_4O_8PS_2$
m/z: 1635 (100.0%), 1633 (86.3%), 1636 (68.3%), 1634 (68.3%),
1637 (33.9%), 1631 (22.6%), 1632 (19.1%), 1638 (13.1%)
Intens.
x10^4
m/z

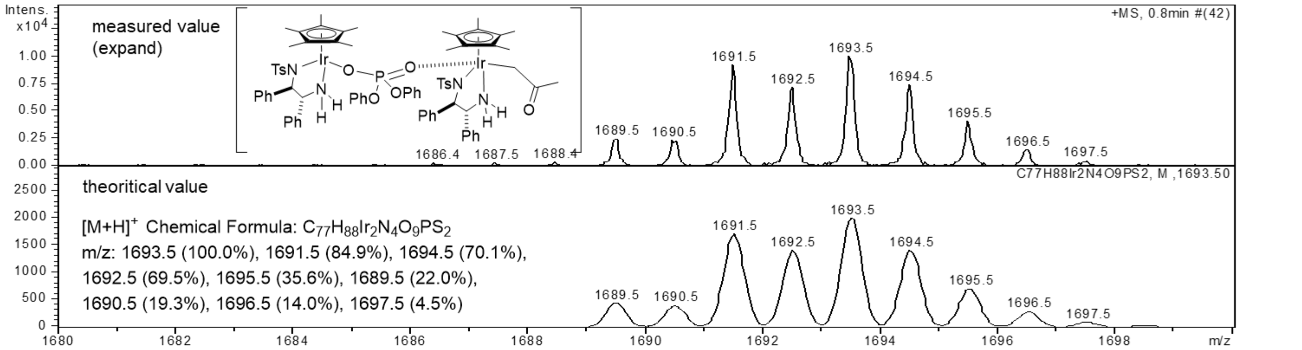
measured value
(expand)
+MS, 0.8min #(42)
TsN
Ir
O
P
O
Ir
Ph
N
H
H
PhO
OPh
TsN
Ph
N
H
H
Ph
O
1686.4
1687.5
1688.4
1689.5
1690.5
1691.5
1692.5
1693.5
1694.5
1695.5
1696.5
1697.5
theoritical value
C77H88Ir2N4O9PS2, M ,1693.50
[M+H]+ Chemical Formula: $C_{77}H_{88}Ir_2N_4O_9PS_2$
m/z: 1693.5 (100.0%), 1691.5 (84.9%), 1694.5 (70.1%),
1692.5 (69.5%), 1695.5 (35.6%), 1689.5 (22.0%),
1690.5 (19.3%), 1696.5 (14.0%), 1697.5 (4.5%)
Intens.
x10^4
m/z

4. Determination of the structure by X-ray Crystallography

The CS Analysis of diol **9**

Diol **3** (in dichloromethane and hexane) was treated with a single crystal of $[(ZnI_2)_3(tpt)_2]$ complex [CS crystal; tpt = 2,4,6-tris(4-pyridyl)triazine], **9** and the guest-absorbed CS crystal was subjected to a diffraction study. ORTEP diagram of the asymmetric unit is shown in Figure S1.

CCDC 2019330 contain the supplementary crystallographic data for this paper. These data can be obtained free of charge from The Cambridge Crystallographic Data Centre via www.ccdc.cam.ac.uk/data_request/cif

Figure S1. Asymmetric unit of the **9**•$[(ZnI_2)_3(tpt)_2]$ inclusion complex. Solvent (dichloromethane) and hydrogens have been removed for clarify.

Figure S2. Diol **9**; ellipsoids are at 50% probability

Experimental. A Single colourless rod-shaped crystals was attached to a capton film on an Rigaku XtaLAB PRO diffractometer. The crystal was kept at a steady T = 100.15 K during data collection. The structure was solved with the **ShelXT** (Sheldrick, 2015) structure solution program using the Intrinsic Phasing solution method and by using **Olex2** (Dolomanov et al., 2009) as the graphical interface. The model was refined with version 2018/3 of **ShelXL** (Sheldrick, 2015) using Least Squares minimisation.

Formula	$C_{56.25}H_{46.5}Cl_{2.5}I_6N_{12}O_4Zn_3$
$D_{calc.}$/ g cm^{-3}	1.703
μ/mm^{-1}	20.865
Formula Weight	2000.69
Colour	colourless
Shape	rod
Size/mm^3	0.15×0.08×0.06
T/K	100.15
Crystal System	monoclinic
Space Group	$P2/c$
a/Å	34.9669(3)
b/Å	14.81360(10)
c/Å	30.7587(2)
α/°	90
β/°	101.5970(10)
γ/°	90
V/Å^3	15607.3(2)
Z	8
Z'	2
Wavelength/Å	1.54184
Radiation type	CuK_α
θ_{min}/°	2.580
θ_{max}/°	80.472
Measured Refl.	320208
Independent Refl.	32630
Reflections with I > 2(I)	25738
R_{int}	0.0879
Parameters	1690
Restraints	923
Largest Peak	1.993
Deepest Hole	-1.209
GooF	1.225
wR_2 (all data)	0.3029
wR_2	0.2857
R_1 (all data)	0.0976
R_1	0.0884

Structure Quality Indicators

**Reflections:**	d min (Cu) 0.78	I/σ 29.0	Rint 8.79%	complete 100% (IUCr) 100%
**Refinement:**	Shift -0.001	Max Peak 2.0	Min Peak -1.2	GooF 1.225

A colorless rod-shaped crystal with dimensions 0.15×0.08×0.06 mm^3 was attached to a Kapton film. Data were collected using a Rigaku XtaLAB PRO diffractometer operating at T = 100.15 K.
Data were measured using ω scans using CuK_α radiation. The total number of runs and images was based on the strategy calculation from the program **CrysAlisPro** (Rigaku, V1.171.40.35a, 2018) The maximum resolution that was achieved was θ = 80.472° (0.78 Å).
The diffraction pattern was indexed. The total number of runs and images was based on the strategy calculation from the program **CrysAlisPro** (Rigaku, V1.171.40.35a, 2018) and the unit cell was refined using **CrysAlisPro** (Rigaku, V1.171.40.35a, 2018) on 97276 reflections, 30% of the observed reflections.
Data reduction, scaling and absorption corrections were performed using **CrysAlisPro** (Rigaku, V1.171.40.35a, 2018). The final completeness is 100.00 % out to 80.472° in θ. A gaussian absorption correction was performed using CrysAlisPro 1.171.40.35a (Rigaku Oxford Diffraction, 2018)Numerical absorption correction based on gaussian integration over a multifaceted crystal model Empirical absorption correction using spherical harmonicsas implemented in SCALE3 ABSPACK. The absorption coefficient μ of this material is 20.865 mm^{-1} at this wavelength (λ = 1.542Å) and the minimum and maximum transmissions are 0.005 and 0.156.
The structure was solved and the space group *P*2/*c* (# 13) determined by the **ShelXT** (Sheldrick, 2015) structure solution program using Intrinsic Phasing and refined by Least Squares using version 2018/3 of **ShelXL** (Sheldrick, 2015). All non-hydrogen atoms were refined anisotropically. Hydrogen atom positions were calculated geometrically and refined using the riding model. Hydrogen atom positions were calculated geometrically and refined using the riding model.
_exptl_absorpt_process_details: CrysAlisPro 1.171.40.35a (Rigaku Oxford Diffraction, 2018)Numerical absorption correction based on gaussian integration over a multifaceted crystal modelEmpirical absorption correction using spherical harmonicsas implemented in SCALE3 ABSPACK.
The value of Z' is 2. This means that there are two independent molecules in the asymmetric unit.
*Crystal structure of (5aS,8aR)-**11***

CCDC 2022569 contain the supplementary crystallographic data for this paper. These data can be obtained free of charge from The Cambridge Crystallographic Data Centre via www.ccdc.cam.ac.uk/data_request/cif
A. Crystal Data

Empirical Formula C19H18O4
Formula Weight 310.35
Crystal Color, Habit colorless, block
Crystal Dimensions 0.136 X 0.082 X 0.077 mm
Crystal System orthorhombic
Lattice Type Primitive
Lattice Parameters a = 8.91118(16) Å
b = 11.0174(2) Å
c = 15.8907(11) Å
V = 1560.12(12) Å3
Space Group P212121 (#19)
Z value 4
Dcalc 1.321 g/cm3
F000 656.00
μ(CuKα) 7.545 cm-1

B. Intensity Measurements

Diffractometer R-AXIS RAPID 191R
Radiation CuKα (λ = 1.54187 Å)
Voltage, Current 45kV, 55mA
Temperature -150.0oC
Detector Aperture 783.0 x 382.0 mm
Data Images 56 exposures
ω oscillation Range (χ=54.0, Φ=0.0) 80.0 - 255.0o
Exposure Rate 3.0 sec./o
ω oscillation Range (χ =54.0, Φ=60.0) 80.0 - 255.0o
Exposure Rate 3.0 sec./o
ω oscillation Range (χ =54.0, Φ=120.0) 80.0 - 255.0o
Exposure Rate 3.0 sec./o
ω oscillation Range (χ =54.0, Φ=180.0) 80.0 - 255.0o
Exposure Rate 3.0 sec./o
ω oscillation Range (χ =54.0, Φ=240.0) 80.0 - 255.0o
Exposure Rate 3.0 sec./o
ω oscillation Range (χ =54.0, Φ=320.0) 80.0 - 255.0o
Exposure Rate 3.0 sec./o

ω oscillation Range (χ =20.0, Φ=0.0) 80.0 - 255.0o
Exposure Rate 3.0 sec./o
ω oscillation Range (χ =20.0, Φ=120.0) 80.0 - 255.0o
Exposure Rate 3.0 sec./o
Detector Position 191.00 mm
Pixel Size 0.100 mm
2θmax 136.5o
No. of Reflections Measured Total: 29839
Unique: 2849 (Rint = 0.0327)
Parsons quotients (Flack x parameter): 1068
Corrections Lorentz-polarization
Absorption
(trans. factors: 0.822 - 0.944)
Secondary Extinction
(coefficient: 8.80000e-004)

C. Structure Solution and Refinement

Structure Solution Direct Methods (SHELXT Version 2014/5)
Refinement Full-matrix least-squares on F2
Function Minimized Σ w (Fo2 - Fc2)2
Least Squares Weights w = 1/ [σ2(Fo2) + (0.0290 . P)2
+ 0.3245 . P]
where P = (Max(Fo2,0) + 2Fc2)/3
2□max cutoff 136.5o
Anomalous Dispersion All non-hydrogen atoms
No. Observations (All reflections) 2849
No. Variables 209
Reflection/Parameter Ratio 13.63
Residuals: R1 (I>2.00σ(I)) 0.0319
Residuals: R (All reflections) 0.0342
Residuals: wR2 (All reflections) 0.0707
Goodness of Fit Indicator 1.090
Flack parameter (Parsons' quotients = 1068) -0.13(6)
Max Shift/Error in Final Cycle 0.000
Maximum peak in Final Diff. Map 0.15 e-/Å3
Minimum peak in Final Diff. Map -0.22 e-/Å3

5. HPLC Chart

(*5aS,8aR*)-3,3-diphenyltetrahydro-1H,6H-furo[3,4-e][1,3]dioxepin-6-one (11)

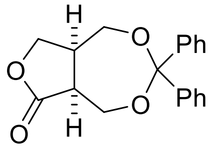

(5a*S*,8a*R*)-**11**

HPLC conditions: DAICEL CHIRALPAK IA-3, hexane/*i*-PrOH = 9/1, flow rate =1.0 mL/min, detection 219 nm, retention time = 9.7 min (5a*R*,8a*S*) and 12.0 min (5a*S*,8a*R*).

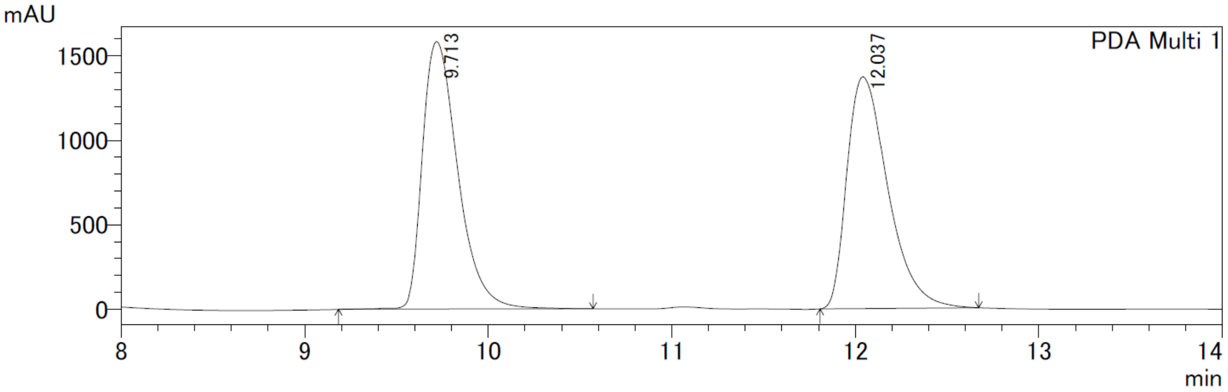

1 PDA Multi 1/219nm 4nm

PDA Ch1 219nm 4nm

Peak#	Ret. Time	Area	Area%
1	9.713	21280689	49.941
2	12.037	21330816	50.059
合計		42611504	100.000

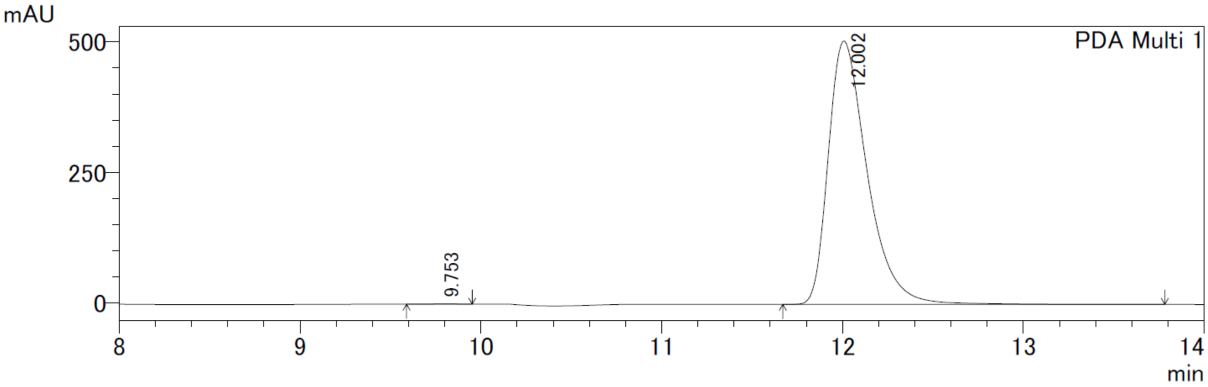

1 PDA Multi 1/219nm 4nm

PDA Ch1 219nm 4nm

Peak#	Ret. Time	Area	Area%
1	9.753	5316	0.070
2	12.002	7548613	99.930
合計		7553929	100.000

cedarmicine A (15a)

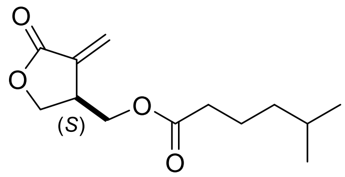

15a:Cedarmycin A

HPLC conditions: DAICEL CHIRALPAK IC-3, hexane/*i*-PrOH = 8/2, flow rate =1.0 mL/min, detection 212 nm, retention time = 23 min (*R*) and 28 min (*S*).

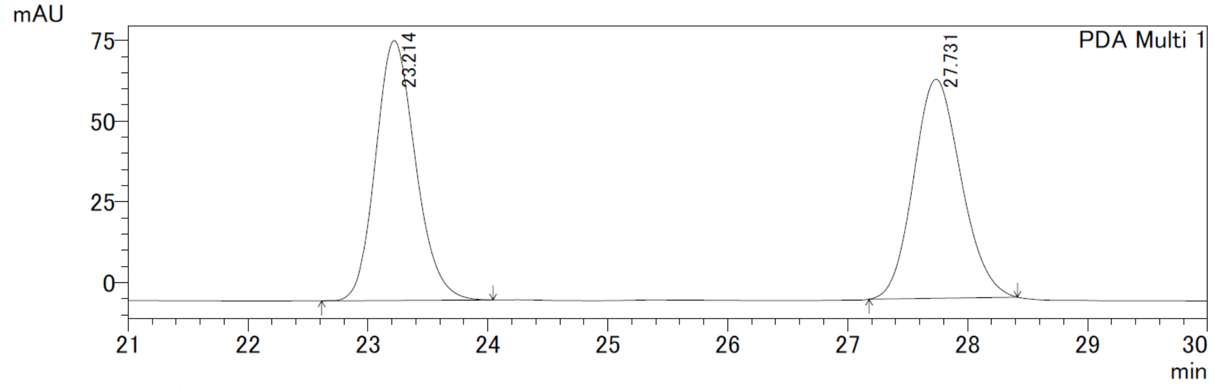

1 PDA Multi 1/212nm 4nm

PDA Ch1 212nm 4nm

Peak#	Ret. Time	Area	Area%
1	23.214	1868099	49.964
2	27.731	1870758	50.036
合計		3738856	100.000

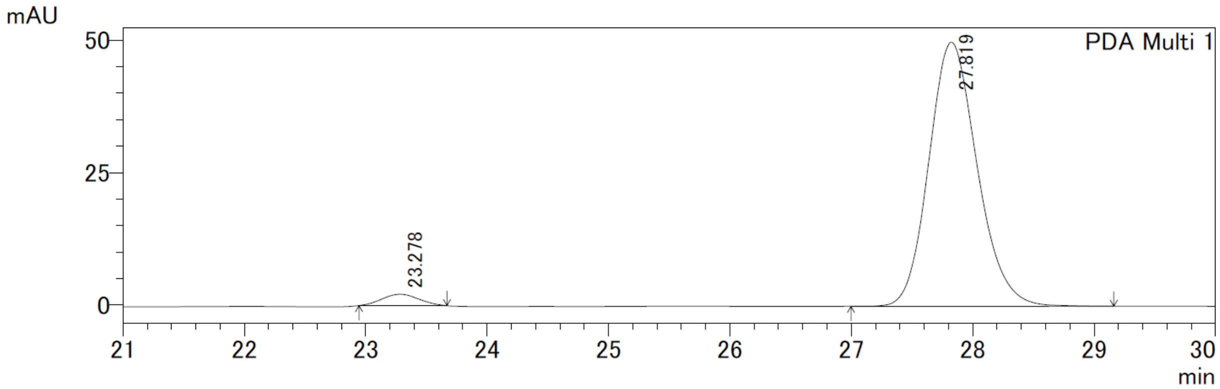

1 PDA Multi 1/212nm 4nm

PDA Ch1 212nm 4nm

Peak#	Ret. Time	Area	Area%
1	23.278	45759	3.215
2	27.819	1377456	96.785
合計		1423215	100.000

Cedarmicine B (15b)

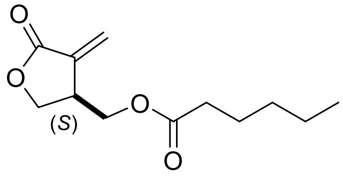

15b: Cedarmycin B

HPLC conditions: DAICEL CHIRALPAK IC-3, hexane/*i*-PrOH = 8/2, flow rate =1.0 mL/min, detection 212 nm, retention time = 31 min (*R*) and 37 min (*S*).

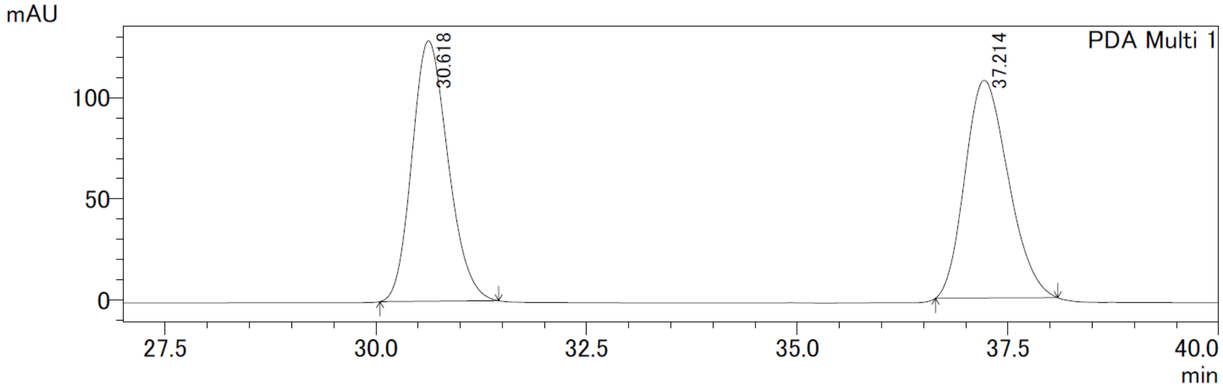

1 PDA Multi 1/212nm 4nm

PDA Ch1 212nm 4nm

Peak#	Ret. Time	Area	Area%
1	30.618	3846776	49.924
2	37.214	3858489	50.076
合計		7705265	100.000

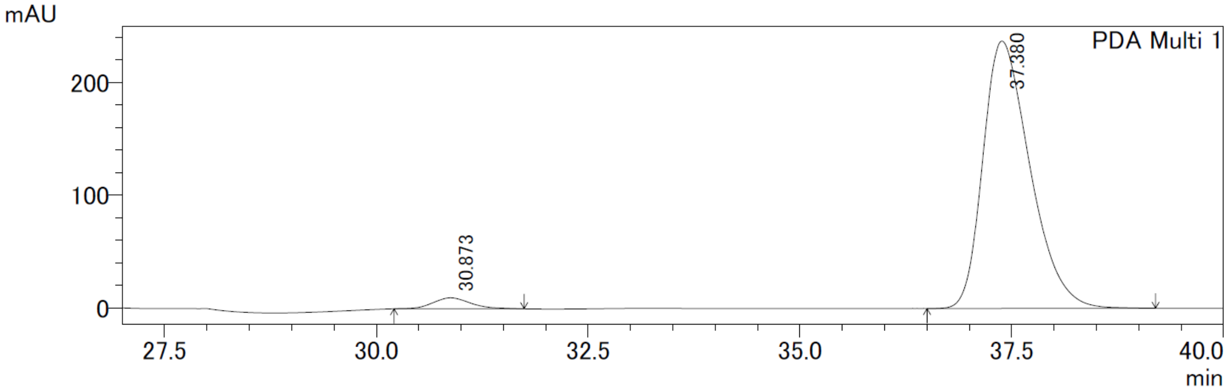

1 PDA Multi 1/212nm 4nm

PDA Ch1 212nm 4nm

Peak#	Ret. Time	Area	Area%
1	30.873	289426	3.062
2	37.380	9162611	96.938
合計		9452037	100.000

6. NMR Spectra

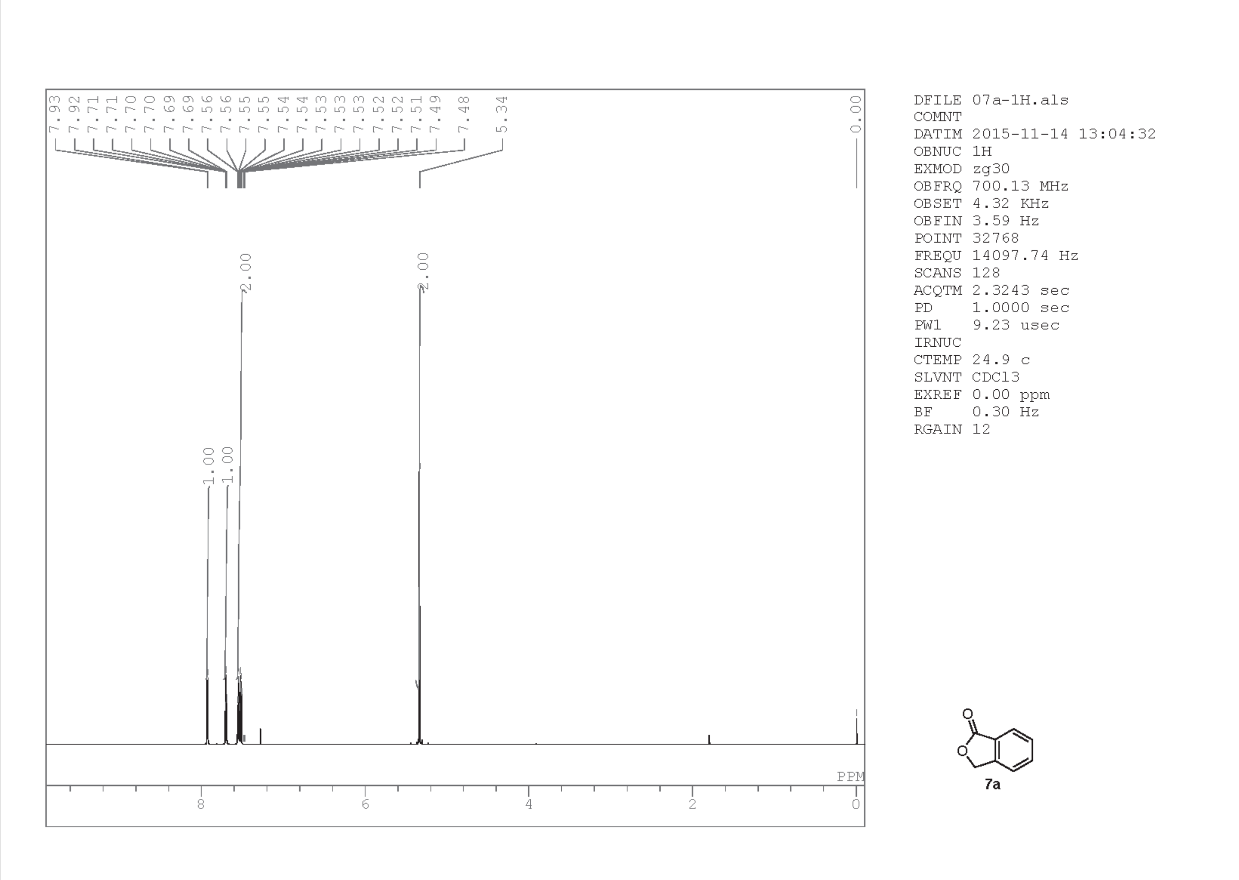

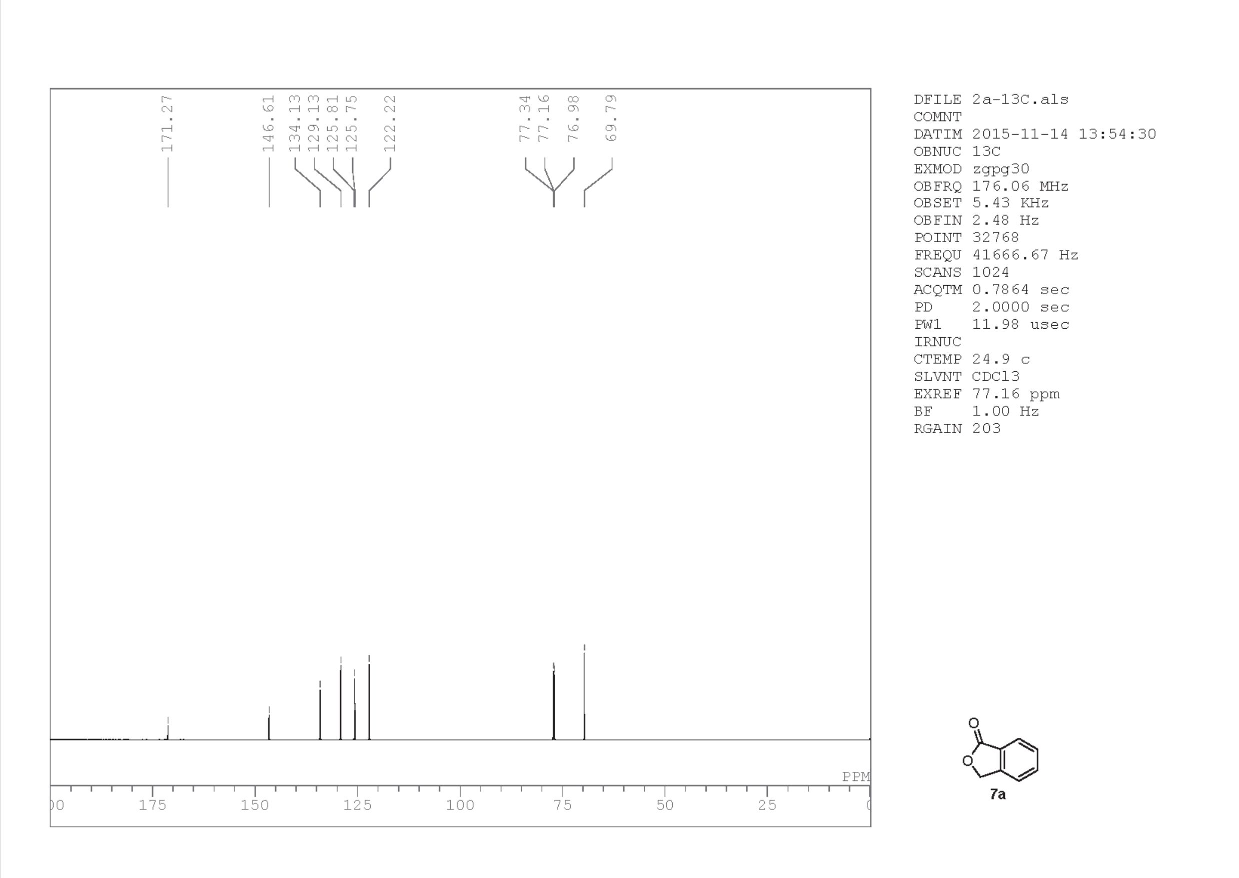

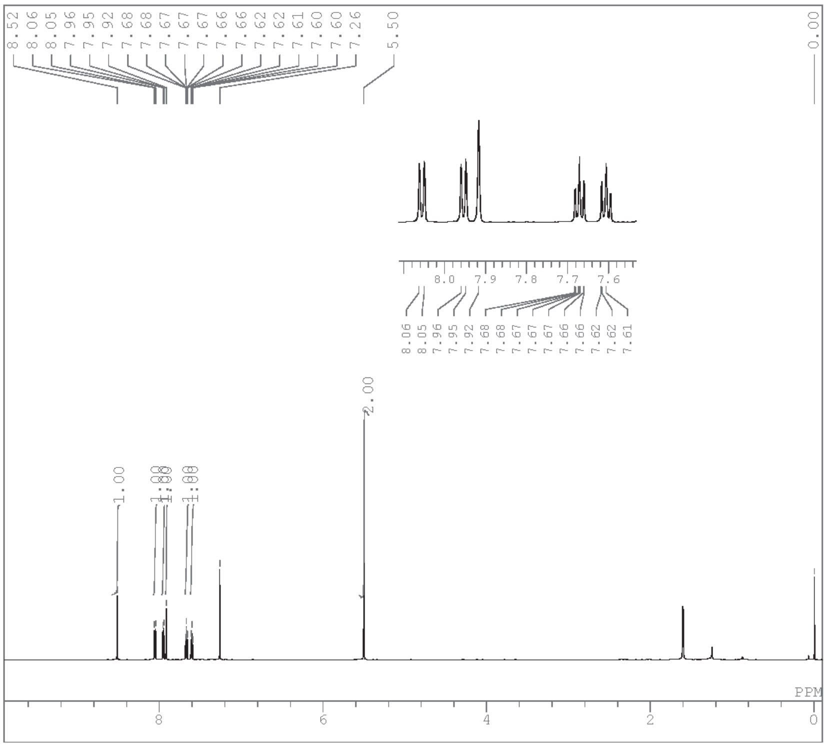

DFILE 07b-1H.als
COMNT
DATIM 2015-11-14 14:05:07
OBNUC 1H
EXMOD zg30
OBFRQ 700.13 MHz
OBSET 4.32 KHz
OBFIN 3.59 Hz
POINT 32768
FREQU 14097.74 Hz
SCANS 128
ACQTM 2.3243 sec
PD 1.0000 sec
PW1 9.23 usec
IRNUC
CTEMP 24.9 c
SLVNT CDCl3
EXREF 0.00 ppm
BF 0.30 Hz
RGAIN 18

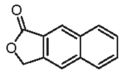

7b

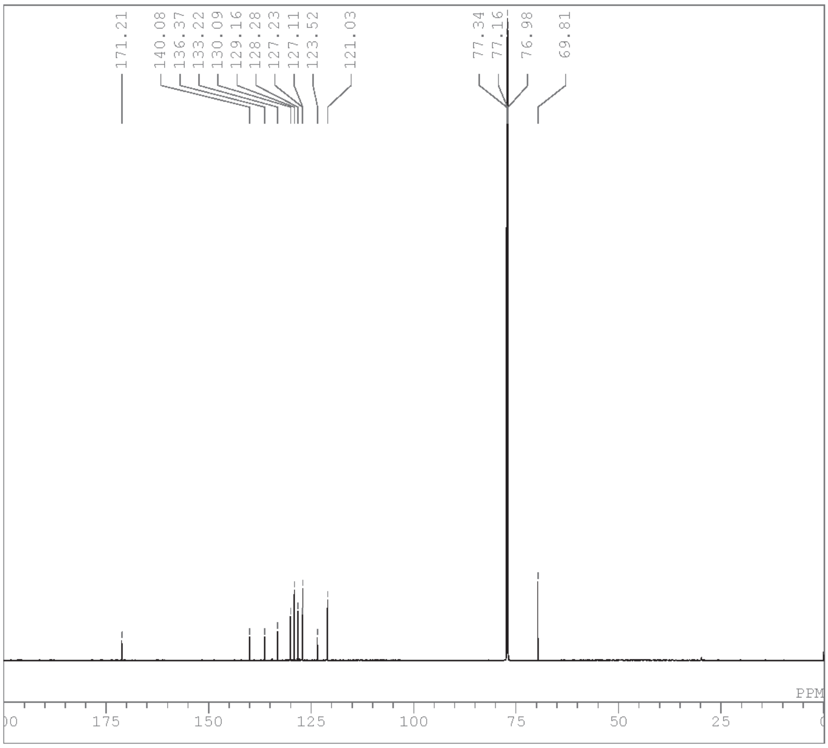

DFILE 2b-13C.als
COMNT
DATIM 2015-11-14 14:54:59
OBNUC 13C
EXMOD zgpg30
OBFRQ 176.06 MHz
OBSET 5.43 KHz
OBFIN 2.48 Hz
POINT 32768
FREQU 41666.67 Hz
SCANS 1024
ACQTM 0.7864 sec
PD 2.0000 sec
PW1 11.98 usec
IRNUC
CTEMP 24.9 c
SLVNT CDCl3
EXREF 77.16 ppm
BF 1.00 Hz
RGAIN 203

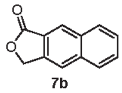

7b

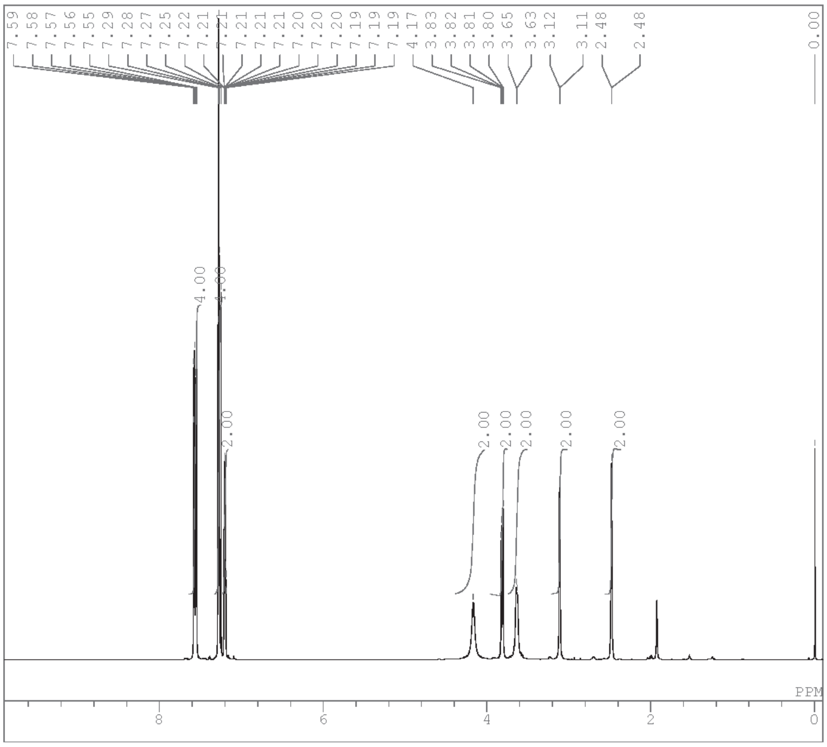

DFILE 09-1H.als
COMNT 1H NMR
DATIM 2015-09-15 22:58:14
OBNUC 1H
EXMOD zg30
OBFRQ 700.13 MHz
OBSET 4.32 KHz
OBFIN 3.59 Hz
POINT 32768
FREQU 14097.74 Hz
SCANS 16
ACQTM 2.3243 sec
PD 1.0000 sec
PW1 9.23 usec
IRNUC
CTEMP 24.9 c
SLVNT CD2Cl2
EXREF 0.00 ppm
BF 0.30 Hz
RGAIN 11

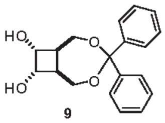

9

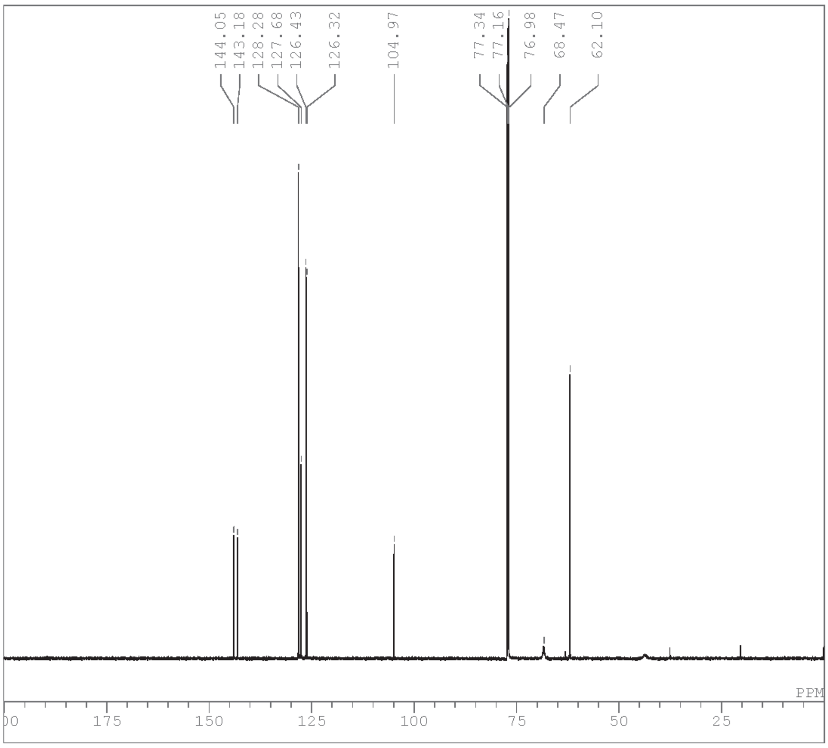

DFILE 09-13C(176).als
COMNT 13C NMR
DATIM 2015-09-15 23:04:51
OBNUC 13C
EXMOD zgpg30
OBFRQ 176.06 MHz
OBSET 5.43 KHz
OBFIN 2.48 Hz
POINT 32768
FREQU 41666.67 Hz
SCANS 128
ACQTM 0.7843 sec
PD 2.0000 sec
PW1 11.98 usec
IRNUC
CTEMP 24.9 c
SLVNT CD2Cl2
EXREF 77.16 ppm
BF 1.00 Hz
RGAIN 203

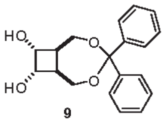

9

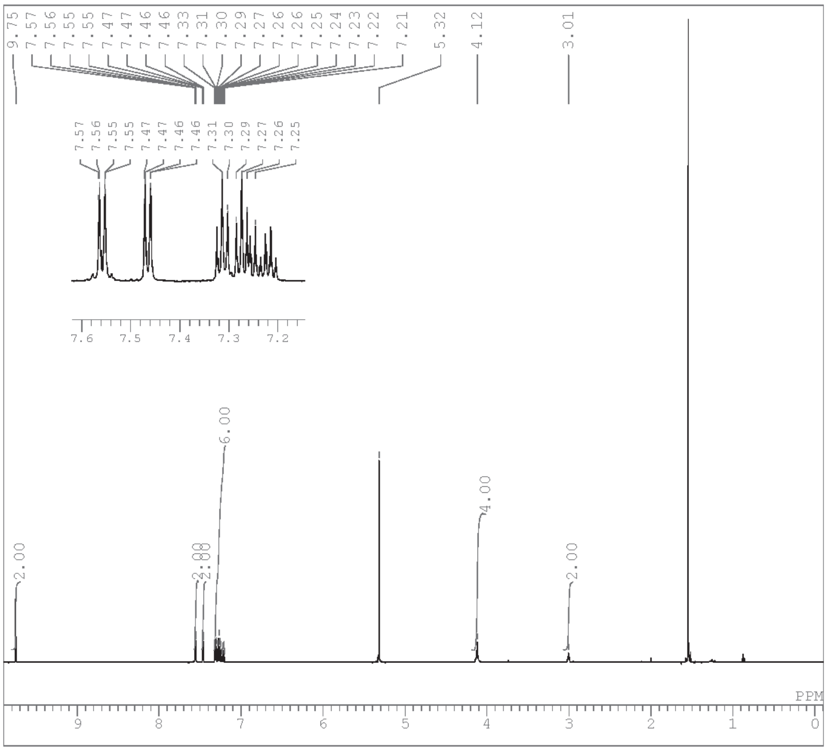

DFILE 10-1H(TD-1029).als
COMNT
DATIM 2015-11-17 06:44:37
OBNUC 1H
EXMOD zg30
OBFRQ 700.13 MHz
OBSET 4.32 KHz
OBFIN 3.59 Hz
POINT 65536
FREQU 14097.74 Hz
SCANS 16
ACQTM 0.0000 sec
PD 0.0000 sec
PW1 10.00 usec
IRNUC
CTEMP 24.9 c
SLVNT CD2Cl2
EXREF 5.32 ppm
BF 0.10 Hz
RGAIN 16

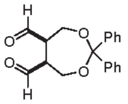

10

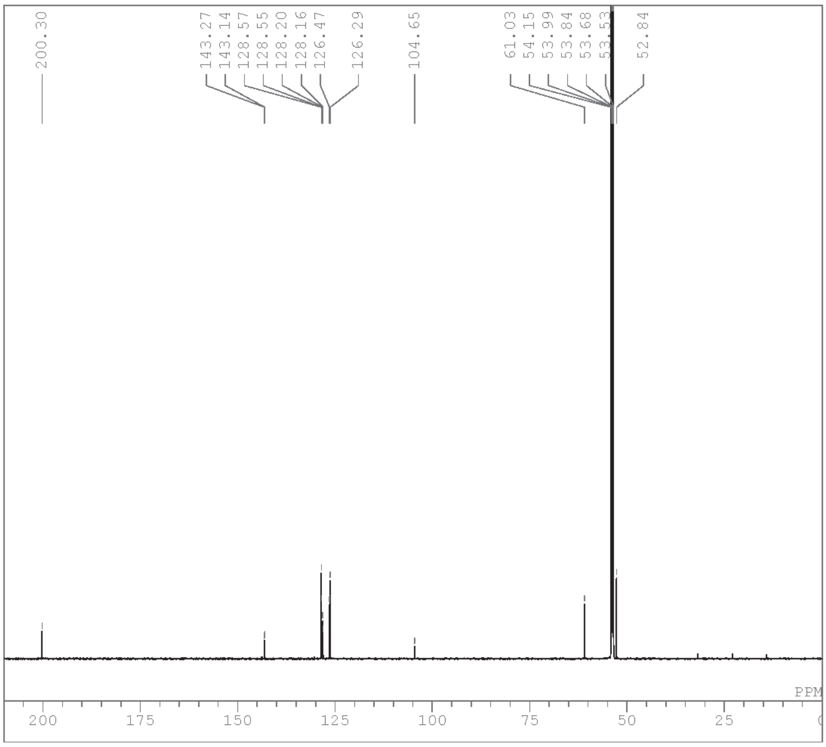

DFILE 10-13C(TD-1029).als
COMNT
DATIM 2015-11-17 14:49:29
OBNUC 13C
EXMOD zgpg30
OBFRQ 176.06 MHz
OBSET 5.43 KHz
OBFIN 2.48 Hz
POINT 32768
FREQU 41666.67 Hz
SCANS 10000
ACQTM 0.0000 sec
PD 0.0000 sec
PW1 10.00 usec
IRNUC
CTEMP 24.9 c
SLVNT CD2Cl2
EXREF 53.84 ppm
BF 1.00 Hz
RGAIN 203

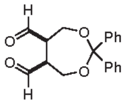

10

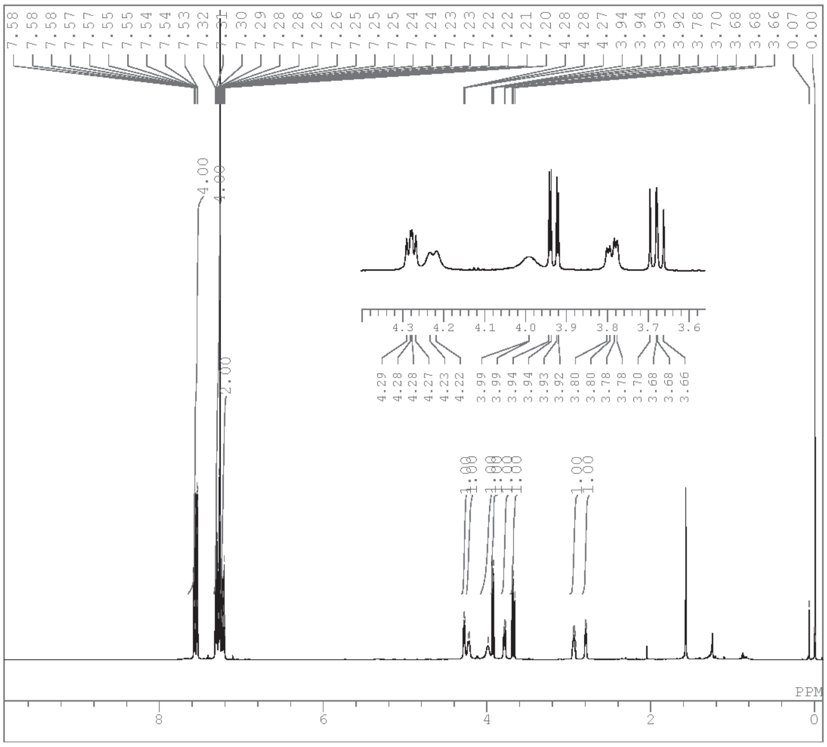

DFILE 11-1H(lactone).als
COMNT 20130409
DATIM 2013-04-09 11:24:14
OBNUC 1H
EXMOD zg30
OBFRQ 700.13 MHz
OBSET 4.32 KHz
OBFIN 3.59 Hz
POINT 32768
FREQU 14097.74 Hz
SCANS 16
ACQTM 2.3243 sec
PD 1.0000 sec
PW1 9.23 usec
IRNUC
CTEMP 24.9 c
SLVNT CDCl3
EXREF 0.00 ppm
BF 0.30 Hz
RGAIN 12

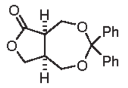

(5a*R*,8a*S*)-**11**

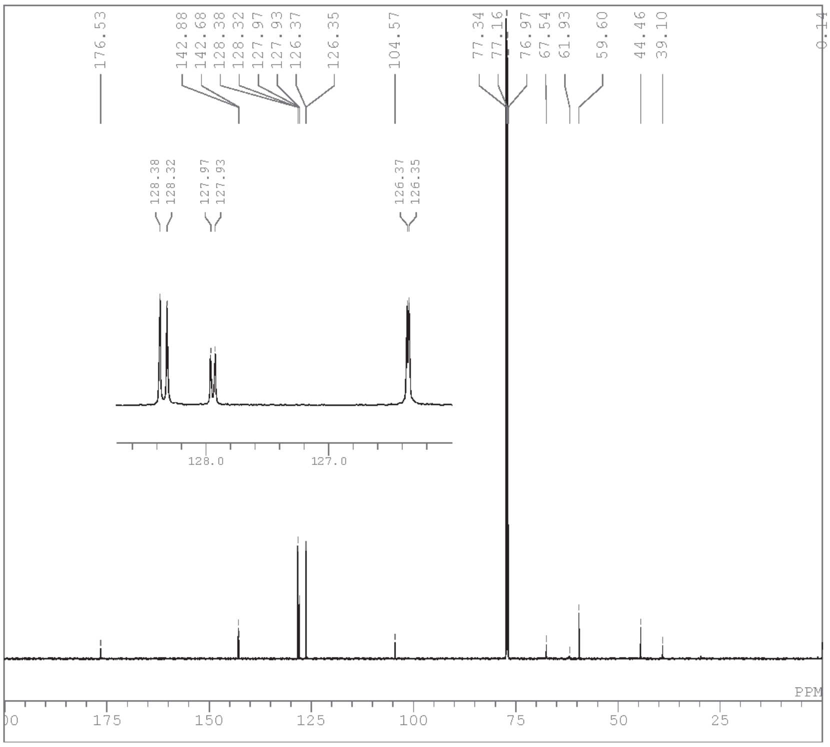

DFILE 11-13C.als
COMNT 20130409
DATIM 2013-04-09 11:50:04
OBNUC 13C
EXMOD zgpg30
OBFRQ 176.06 MHz
OBSET 5.43 KHz
OBFIN 2.48 Hz
POINT 32768
FREQU 41666.67 Hz
SCANS 512
ACQTM 0.7843 sec
PD 2.0000 sec
PW1 11.98 usec
IRNUC
CTEMP 24.9 c
SLVNT CDCl3
EXREF 77.16 ppm
BF 1.00 Hz
RGAIN 203

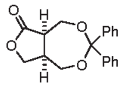

(5a*R*,8a*S*)-**11**

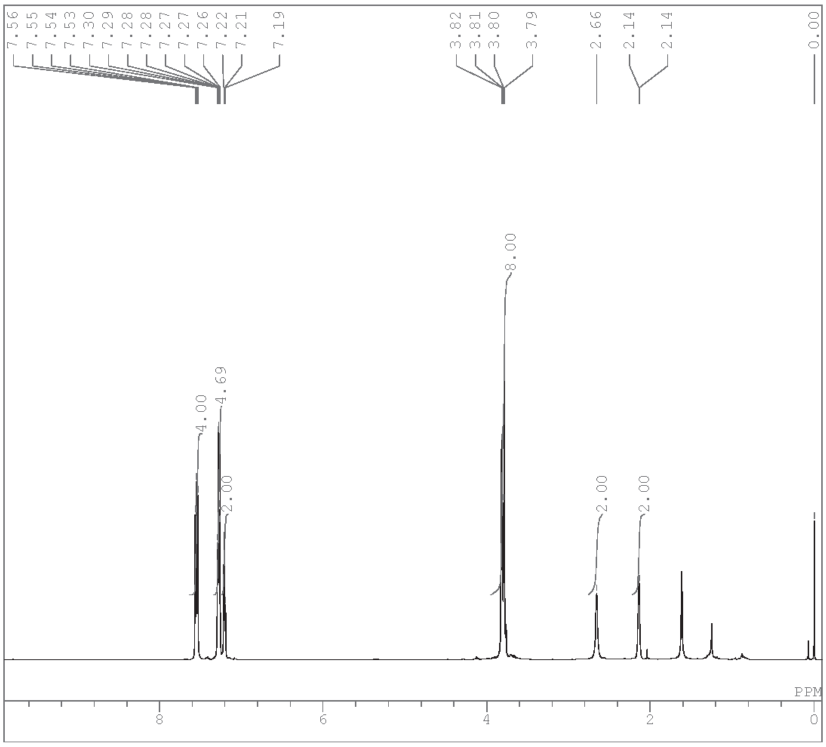


```
DFILE 12-1H(I-06-198-F2-3_Proton-
COMNT I-06-198-F2-3
DATIM 2015-10-23 15:12:56
OBNUC 1H
EXMOD proton.jxp
OBFRQ 600.17 MHz
OBSET 5.30 KHz
OBFIN 5.47 Hz
POINT 13107
FREQU 9009.01 Hz
SCANS 16
ACQTM 1.4549 sec
PD    5.0000 sec
PW1   9.00 usec
IRNUC 1H
CTEMP 20.0 c
SLVNT CDCL3
EXREF 0.00 ppm
BF    1.00 Hz
RGAIN 44
```

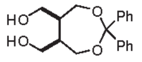

12

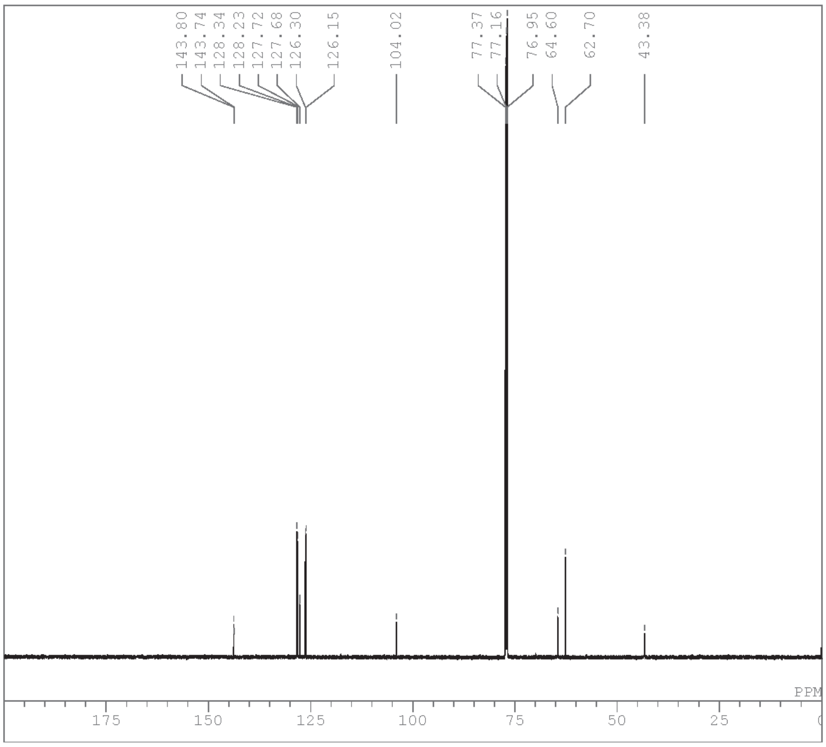


```
DFILE 12-13C(I-06-198-F2-3_Protor
COMNT single pulse decoupled gate
DATIM 2015-10-23 15:14:53
OBNUC 13C
EXMOD carbon.jxp
OBFRQ 150.92 MHz
OBSET 8.52 KHz
OBFIN 1.74 Hz
POINT 26214
FREQU 37878.79 Hz
SCANS 1024
ACQTM 0.6921 sec
PD    2.0000 sec
PW1   3.53 usec
IRNUC 1H
CTEMP 20.5 c
SLVNT CDCL3
EXREF 77.16 ppm
BF    1.00 Hz
RGAIN 50
```

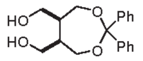

12

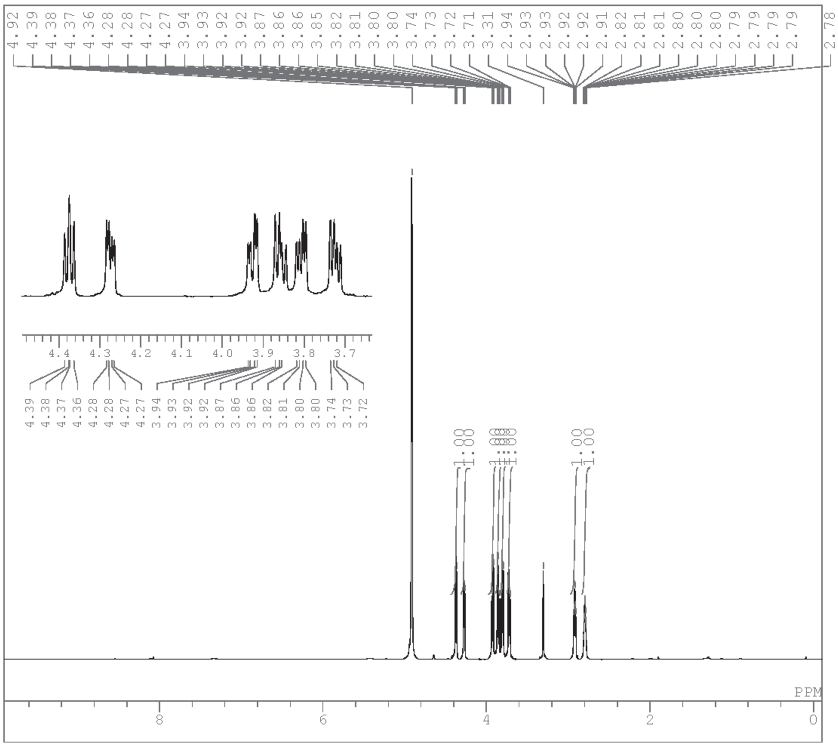

DFILE 14-cis-1H(YA1367f11-13).als
COMNT YA-1367f11-13
DATIM 2019-03-05 15:08:29
OBNUC 1H
EXMOD zg30
OBFRQ 700.13 MHz
OBSET 4.20 KHz
OBFIN 0.78 Hz
POINT 32768
FREQU 10504.20 Hz
SCANS 16
ACQTM 3.1195 sec
PD 1.0000 sec
PW1 9.23 usec
IRNUC
CTEMP 24.9 c
SLVNT MeOD
EXREF 3.31 ppm
BF 0.30 Hz
RGAIN 11

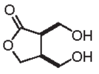

cis-**14**

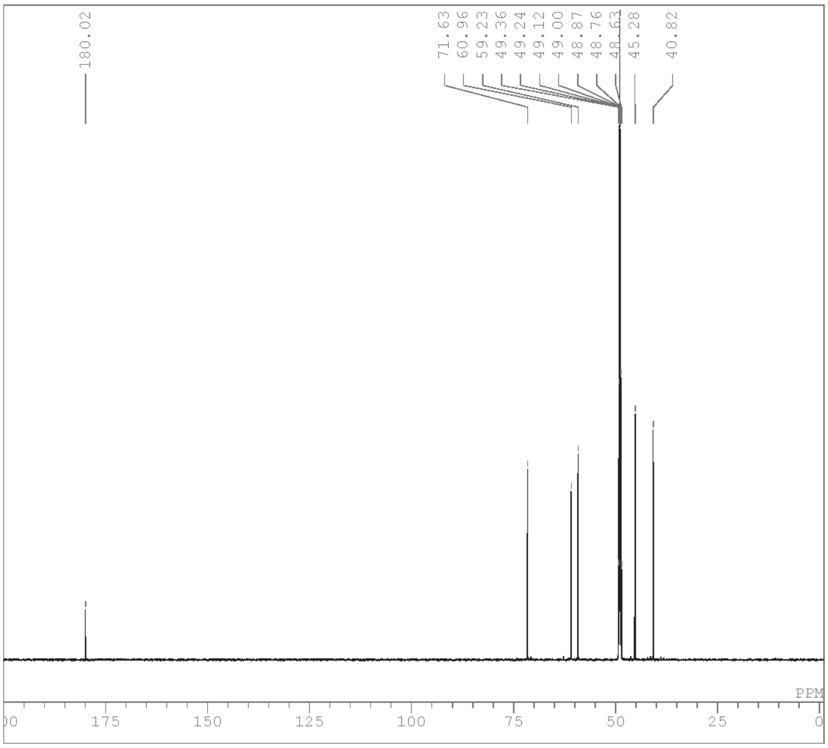

DFILE YA1367f11-13.als
COMNT YA-1367f11-13
DATIM 2019-03-05 15:33:10
OBNUC 13C
EXMOD zgpg30
OBFRQ 176.06 MHz
OBSET 5.43 KHz
OBFIN 3.31 Hz
POINT 32768
FREQU 42613.64 Hz
SCANS 512
ACQTM 0.7668 sec
PD 2.0000 sec
PW1 11.98 usec
IRNUC
CTEMP 24.9 c
SLVNT MeOD
EXREF 49.00 ppm
BF 1.00 Hz
RGAIN 203

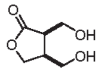

cis-**14**

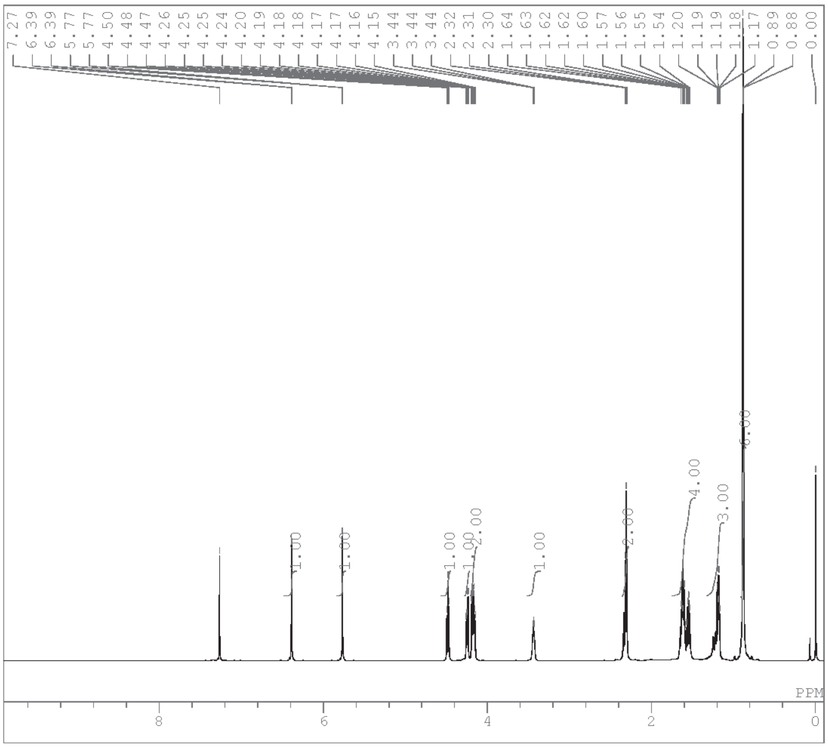

DFILE I-06-041-PURE_Proton-1-1.a
COMNT single_pulse
DATIM 2015-04-18 15:22:20
OBNUC 1H
EXMOD proton.jxp
OBFRQ 600.17 MHz
OBSET 5.30 KHz
OBFIN 5.47 Hz
POINT 13107
FREQU 9009.01 Hz
SCANS 16
ACQTM 1.4549 sec
PD 5.0000 sec
PW1 5.80 usec
IRNUC 1H
CTEMP 17.3 c
SLVNT CDCL3
EXREF 0.00 ppm
BF 1.00 Hz
RGAIN 40

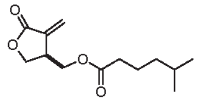

15a:CedarmycinA

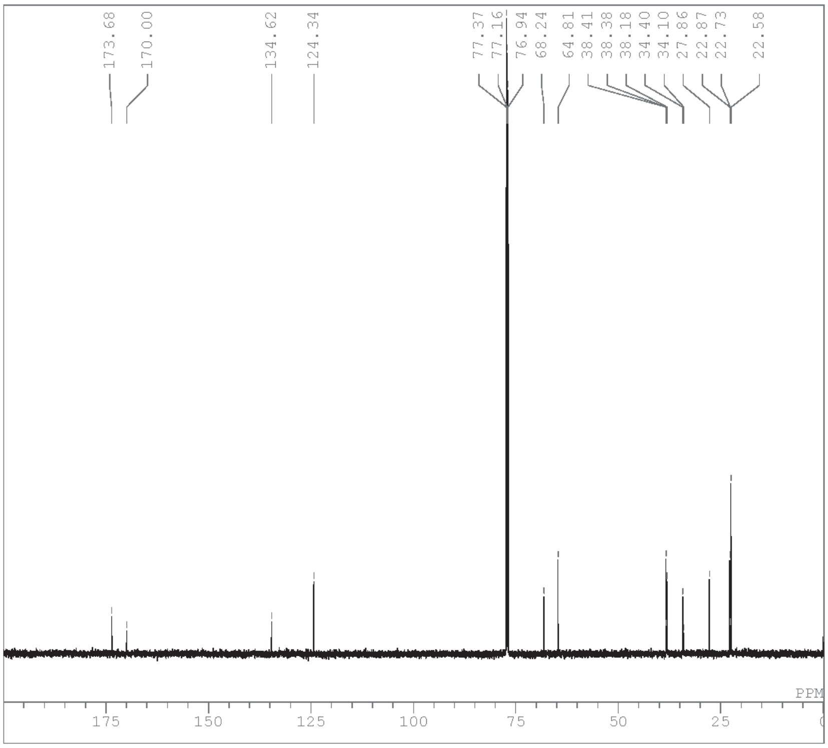

DFILE I-06-041-PURE_Carbon-1-1(9
COMNT single pulse decoupled gat
DATIM 2015-04-18 15:35:47
OBNUC 13C
EXMOD carbon.jxp
OBFRQ 150.92 MHz
OBSET 8.52 KHz
OBFIN 1.74 Hz
POINT 26214
FREQU 37878.79 Hz
SCANS 1024
ACQTM 0.6921 sec
PD 2.0000 sec
PW1 4.60 usec
IRNUC 1H
CTEMP 18.4 c
SLVNT CDCL3
EXREF 77.16 ppm
BF 1.00 Hz
RGAIN 50

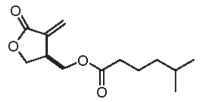

15a:CedarmycinA

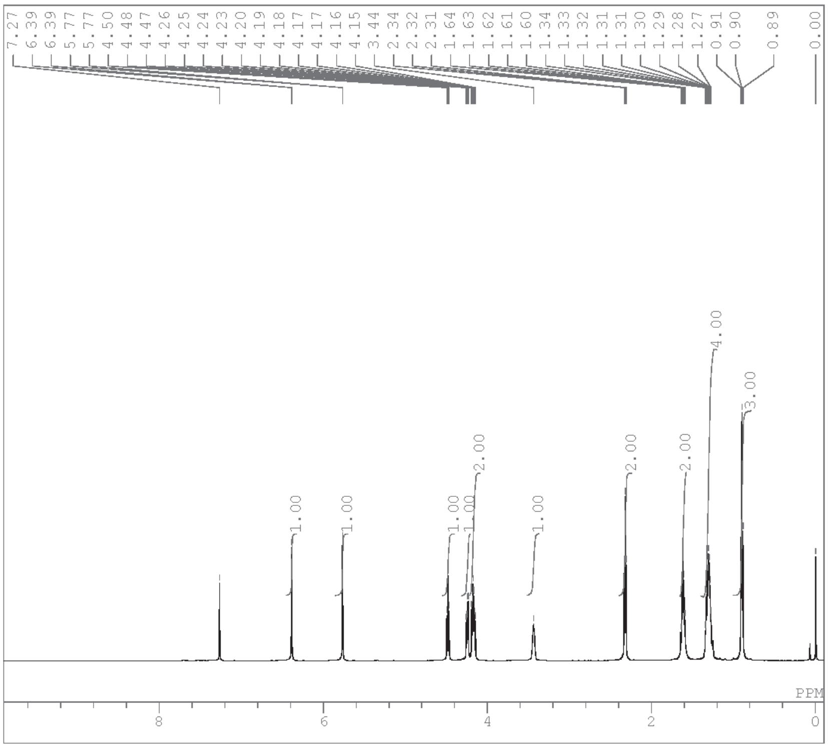

DFILE I-05-146-PURE 2_Proton-1-1
COMNT single_pulse
DATIM 2015-01-20 22:57:34
OBNUC 1H
EXMOD proton.jxp
OBFRQ 600.17 MHz
OBSET 5.30 KHz
OBFIN 5.47 Hz
POINT 13107
FREQU 9009.01 Hz
SCANS 16
ACQTM 1.4549 sec
PD 5.0000 sec
PW1 6.50 usec
IRNUC 1H
CTEMP 16.9 c
SLVNT CDCL3
EXREF 0.00 ppm
BF 1.00 Hz
RGAIN 46

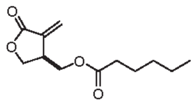

15b: Cedarmycin B

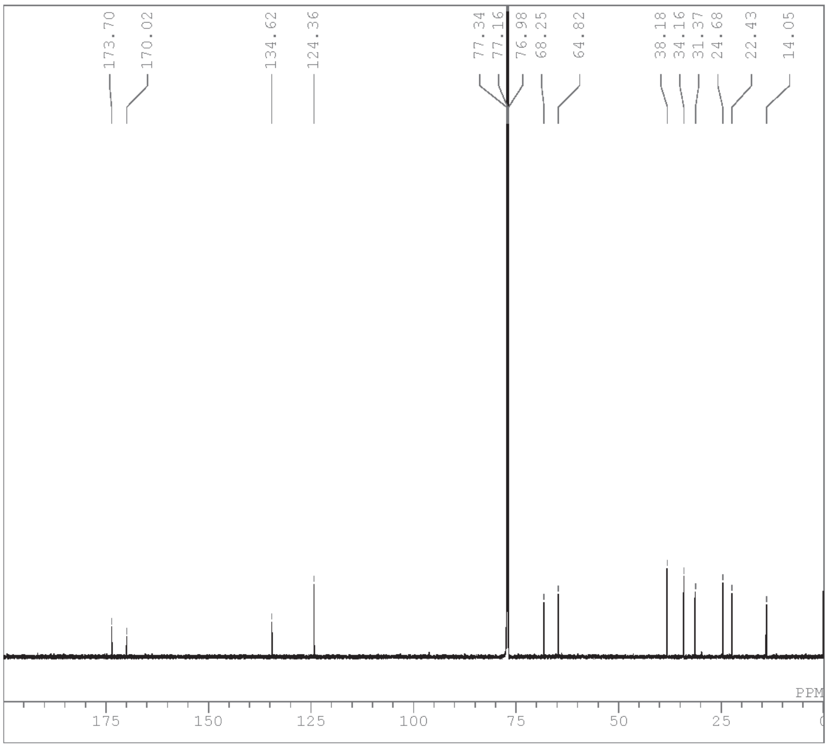

DFILE 1r
COMNT
DATIM 2015-03-17 23:09:43
OBNUC 13C
EXMOD zgpg30
OBFRQ 176.06 MHz
OBSET 5.43 KHz
OBFIN 2.48 Hz
POINT 32768
FREQU 41666.67 Hz
SCANS 1024
ACQTM 0.0000 sec
PD 0.0000 sec
PW1 10.00 usec
IRNUC
CTEMP 24.9 c
SLVNT CDCl3
EXREF 77.16 ppm
BF 0.10 Hz
RGAIN 203

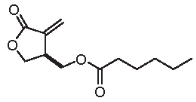

15b: Cedarmycin B
